# Antibody to CMRF35-Like Molecule 2, CD300e A Novel Biomarker Detected in Patients with Fulminant Type 1 Diabetes

**DOI:** 10.1371/journal.pone.0160576

**Published:** 2016-08-11

**Authors:** Fumitaka Haseda, Akihisa Imagawa, Hiroyoshi Nishikawa, Shinobu Mitsui, Chiharu Tsutsumi, Reiko Fujisawa, Hiroyuki Sano, Yuko Murase-Mishiba, Jungo Terasaki, Shimon Sakaguchi, Toshiaki Hanafusa

**Affiliations:** 1 Department of Internal Medicine (I), Osaka Medical College, Takatsuki, Japan; 2 Department of Metabolic Medicine, Graduate School of Medicine, Osaka University, Suita, Japan; 3 Experimental Immunology, Immunology Frontier Research Center, Osaka University, Suita, Japan; 4 Division of Cancer Immunology, Exploratory Oncology Research and Clinical Trial Center, National Cancer Center, Kashiwa, Japan; Baylor College of Medicine, UNITED STATES

## Abstract

**Aims/Hypothesis:**

Fulminant type 1 diabetes (FT1D) is a distinct subtype of type 1 diabetes and is fatal without immediate diagnosis and treatment. At present, there are no biomarkers for early and predictive detection of FT1D.

**Methods:**

First, we analyzed a total of 6 serum samples from 3 patients with FT1D (1 sample in the acute and 1 in the sub-acute phases from each patient) by seromic analysis. Second, titres of the antibody were measured by ELISA in sera from 30 patients with FT1D (both in the acute and sub-acute phases), 13 patients with FT1D in the chronic phase, 32 patients with autoimmune type 1 (type 1A) diabetes (T1AD), 30 patients with type 2 diabetes (T2D), 23 patients with autoimmune thyroid disease (AITD) and 31 healthy control subjects (HC).

**Results:**

Seromic analysis revealed 9 antibodies which showed high signals from all 3 patients with FT1D in the acute phase. Among them, the titre of anti-CD300e antibody was significantly higher in FT1D patients in the acute phase than that in T1AD, T2D, AITD patients and HC, as determined by ELISA (P<0.01, respectively). The titre of anti-CD300e antibody was also higher in FT1D in the acute phase than that in the sub-acute phase (P = 0.0018, Wilcoxon signed-rank test). The titre of anti-LGALS3 antibody in FT1D patients in the acute phase did not differ from that in patients with FT1D in the sub-acute phase, T1AD, T2D, AITD and HC.

**Conclusion/Interpretation:**

The titre of a novel antibody, anti-CD300e, was high in sera from patients with FT1D. This antibody might be a diagnostic marker and provide new insight into the pathogenesis of FT1D.

## Introduction

Fulminant type 1 diabetes (FT1D) is a distinct subtype of type 1 diabetes (T1D) characterized by a rapid onset and an insulin deficiency resulting from almost complete destruction of pancreatic beta cells even at the disease onset [1, 2]. A nationwide survey identified that this variant accounts for 19.4% of acute-onset T1D patients in Japan [2]. Many cases have been reported from other countries, especially in East Asia [3–6]. Because of the remarkably abrupt onset and very short duration (generally less than 1 week) of diabetic symptoms, which indicate a remarkably rapid destruction of pancreatic beta cells, this subtype would be fatal without immediate diagnosis and treatment. However, we currently have no appropriate biomarker to diagnose this subtype that is equivalent to the islet-cell antibodies (ICA), anti-glutamic acid decarboxylase (GAD) antibodies, insulin autoantibodies, anti-insulinoma associated antigen 2 (IA-2) antibodies and anti-zinc transporter 8 (ZnT8) antibodies used for the diagnosis of autoimmune type 1 (type 1A) diabetes (T1AD) [7–11].

Massive cellular infiltration of T-cells and macrophages has been detected in islets and exocrine pancreas just after disease onset of FT1D [12, 13]. Increased CD4^+^ T-cell responses against GAD, as detected by enzyme-linked immunospot (ELISPOT) assay, have been proposed [14]. Recently, we have reported that CD4^+^CD45RA^-^Foxp3^hi^ activated regulatory T-cells, which play a central role in the T-cell mediated immune response, are functionally impaired both in patients with FT1D and in patients with T1AD [15]. These findings suggest that both innate and acquired immune disorders might contribute to the development of FT1D.

Serum autoantibodies represent an easily accessible surrogate for measuring adaptive immune responses to antigens and might serve as useful diagnostic biomarkers. Gnjatic et al have established “seromic analysis”, which assesses the binding of IgG antibodies against a panel of more than 8000 human antigens by using protein microarrays and fluorescence detection [16, 17]. Recently, novel antibodies, i.e., anti-EEF1A1 and UBE2L3 antibodies, have been detected in patients with T1D by using the seromic analysis [18]. A novel autoantibody to claudin-1 has also been detected in patients with Behçet's disease by using this method [19]. Given the availability of such a new technology, seromic analysis, we explored to discover a novel diagnostic marker in FT1D.

## Methods

### Participants

First, we analyzed a total of 6 serum samples from 3 patients with FT1D (1 sample in the acute and 1 in the sub-acute phases from each patient) on 9418 human protein arrays (Invitrogen ProtoArray Human Protein Microarray v5.0, Carlsbad, CA, USA) by fluorescence (Table 1). All 3 patients with FT1D possessed HLA-DR4, which was most common in FT1D. In this study, we defined the acute and the sub-acute phases of FT1D as less than 2 weeks and from 2 weeks to 2 months after the onset, respectively. We also defined the chronic phase of FT1D as greater than 1 year after the onset. Second, titres of the antibody were measured by ELISA in sera from 30 patients with FT1D (both in the acute and the sub-acute phases, 26 patients for the anti-CD300e antibody assay and 16 patients for the anti-LGALS3 antibody assay), 13 patients with FT1D in the chronic phase, 32 patients with T1AD, 30 patients with type 2 diabetes (T2D), 22 patients with autoimmune thyroid disease (AITD) and 31 healthy control subjects (HC) (Table 2). The serum samples were obtained from fresh whole blood via centrifugal separation and then immediately cryopreserved at -30°C. Patients with FT1D were diagnosed according to the criteria of the Japan Diabetes Society [20]: (1) Occurrence of diabetic ketosis or ketoacidosis soon (around 7 days) after the onset of hyperglycemic symptoms (elevation of urinary or serum ketone bodies at first visit); (2) Plasma glucose level > 288 mg/dl (16.0 mmol) and hemoglobin A1c level < 8.7% (NGSP value) at first visit; (3) Urinary C-peptide excretion < 10 μg/day or fasting serum C-peptide level < 0.3 and < 0.5 ng/ml after intravenous glucagon (or meal) load at onset. All patients with T1AD were GAD or IA-2 antibody positive. Patients with AITD were positive for anti-thyroglobulin (TG) antibody, anti-thyroid peroxidase (TPO) antibody or thyrotropin receptor antibody (TRAb).

**Table 1:** Clinical characteristics of 3 patients with fulminant type 1 diabetes in seromic analysis.

							Duration
	Age	Gender	HbA1c	HbA1c	Glucose	GAD/IA-2	of diabetes
	(years)*	(m/f)†	(%)	(mmol/mol)	(mg/dl)*	antibody	(days)#
Case 1	57	m	6.9	51.9	1275	**−/−**	8/40
Case 2	64	m	7.6	59.5	1015	**−/−**	2/32
Case 3	25	f	5.9	41.0	589	**−/−**	3/28

*at onset

†m/f; male/female

#acute/sub-acute phase

**Table 2:** Clinical characteristics of the subjects.

				GAD/IA-2				Fasting
		Age	Gender	antibody positive	Duration of	HbA1c	HbA1c	C-peptide
Type of disease	n	(years)	(male/female)	(%)	disease*	(%)	(mmol/mol)	(ng/mL)
Fulminant type 1 diabetes								
Acute phase	30	48 (8–82)	24/6#	0/0	6.8±3.2 days†	6.7±0.7	49.8±7.8	0.13±0.14
Sub-acute phase	30				24.2±8.8 days††			
Chronic phase	13	52 (27–73)	8/5	31.0/0	6.9±3.1 years	7.7±0.5	60.7±5.4	0.03±0.07
Type 1A diabetes	32	44 (18–80)	11/21	90.6/52.6 (n = 19)	10.9±9.8 years	7.8±1.6	61.8±17.4	0.29±0.45
Type 2 diabetes	30	56 (29–70)	18/12	0/−	n.d.	7.8±1.6	61.8±17.4	−
Autoimmune thyroid disease	22	50 (18–72)	6/16	−	n.d.	−	−	−
Healthy control subjects	31	39 (25–60)	19/12	−	−	−	−	−

Data are medians (range) or mean ± SD. n.d.: not determined.

*Duration from the onset of diabetes to the time of sample collection.

#P<0.05 (versus type 1A diabetes or autoimmune thyroid disease). P-values were calculated by Fisher’s exact probability test.

†, ††Duration of fulminant type 1 diabetes in acute and sub-acute phase represents days after onset.

Ethical committees at Osaka Medical College approved all study protocols, and written informed consent was obtained from each subject.

### Seromic analysis

ProtoArray^®^ Human Protein Microarrays Ver5 (Invitrogen) were purchased and used according to the manufacturer’s instructions. In brief, the arrays were blocked with a synthetic blocking agent, and 10 μl of patient serum diluted 1:500 in washing buffer [0.1% Tween 20, 1% bovine serum albumin (BSA) in phosphate-buffered saline (PBS)] was added. The arrays were then washed, and Alexa Fluor^®^ 647 dye-labeled goat anti-human IgG was added for detection. After washed and dried, the arrays were scanned using a microarray scanner (Axon 4200A with GenePix Pro Analysis Software, Molecular Devices, Sunnyvale, CA, USA).

### Quantification of CD300e and LGALS3 (galectin-3) autoantibodies by ELISA

Ninety-six well flat-bottom plates (Nunc-Immuno^TM^ MicroWell^TM^ 96 well solid plate, Sigma-Aldrich, St. Louis, MO, USA) were coated with human CD300e recombinant protein (protein accession number: AAI00889.1, Abnova, Waterloo, NSW, Australia) or LGALS3 (galectin-3) (PeproTech, Rocky Hill, NJ, USA) human recombinant protein at 5 ng/50 μl (PBS)/well concentration and incubated overnight at 4°C. After being washed, the plates were blocked with 1% BSA (200 μl/ well) (Sigma-Aldrich) for 1 hour. The plates were then washed, and 100 μl/well of diluted patient serum was added (1:100 diluted by 1% BSA in PBS) in duplicate and incubated for 2 hours. After being washed, anti-human IgG-HRP (horseradish peroxidase) goat IgG/Fab' (MBL, Woburn, MA, USA) (1:4000) in 1% BSA was added (100 μl/well) and incubated for 2 hours at 4°C in the dark. The plates were washed and then incubated with 100 μl of TMB (3,3’,5,5’-tetramethylbenzidine) substrate solution (Thermo, Rockford, IL, USA) for 7 minutes at room temperature. The reaction was terminated by adding 100 μl of 0.18 M H_2_SO_4_ (Wako, Osaka, Japan), and the absorbance was determined at an optical density of 450 nm. The coefficient of variation (CV) among the assays was less than 10.0%.

### Specificity analysis of ELISAs by flow cytometry

HEK293 cells were purchased from DS Pharma Biomedical (Osaka, Japan) (cell line: 2931, accession number: 85120602), and cultured in E-MEM medium (DS Pharma Biomedical) supplemented with 1% non-essential amino acids (DS Pharma Biomedical), 10% fetal bovine serum (FBS) (DS Pharma Biomedical), 2mM glutamic acid (DS Pharma Biomedical) and 10 ml/l penicillin-streptomycin solution (DS Pharma Biomedical) in a 6-well plate. The cell density of HEK293 at transfection was > 80% confluence. Plasmid DNA (pBApo-CMV Neo DNA) integrated with the CD300e gene (TAKARA BIO INC, Shiga, Japan) was transfected using TransIT-293 Transfection Reagent (Mirus Bio LLC, Madison WI, USA), according to the manufacturer’s recommendations.

CD300e-transfected HEK293 cells were harvested and immediately subjected to cellular staining. The cells were added with 20 μl of sera (1×) from 17 subjects (10 from FT1D in the acute phase and 7 from HC selected randomly), incubated for 1 h at room temperature, washed twice with PBS supplemented with 2% FBS, and then stained with secondary phycoerythrin (PE)-mouse anti-human immunoglobulin (Ig) G (BD Bioscience, San Jose, CA, USA) for 30 min. As a positive or negative control, the cells were stained with PE anti-human CD300e (clone UP-H2) (BioLegend, San Diego, CA, USA) or PE mouse IgG1 κ isotype control (BD Bioscience). The stained cells were then subjected to flow cytometric analysis using a BD FACSAria^TM^ Cell Sorter (BD Bioscience). We first gated on CD300e-transfected HEK293 cells and then counted the number of target cells. BD FACSDiva Software was used for analysis of the cytometric data. We defined the target cell populations based on 0.5% of isotype controls. At least 50,000 CD300e-transfected HEK293 cells were acquired from each sample.

### Statistical analysis

Statistical analyses were carried out using the Mann-Whitney *U*-test, two-tailed unpaired Student’s *t*-test, Wilcoxon signed-rank test and receiver operating characteristic (ROC) analysis (JMP^®^ Ver.10, SAS Institute Japan and GraphPad Prism 6 Software, La Jolla, CA, USA). Pearson correlation was used to calculate the r value (GraphPad Prism 6 Software). For all tests, a p value of <0.05 was considered to be statistically significant.

## Results

### Seromic analysis in patients with FT1D

**Fig 1:**
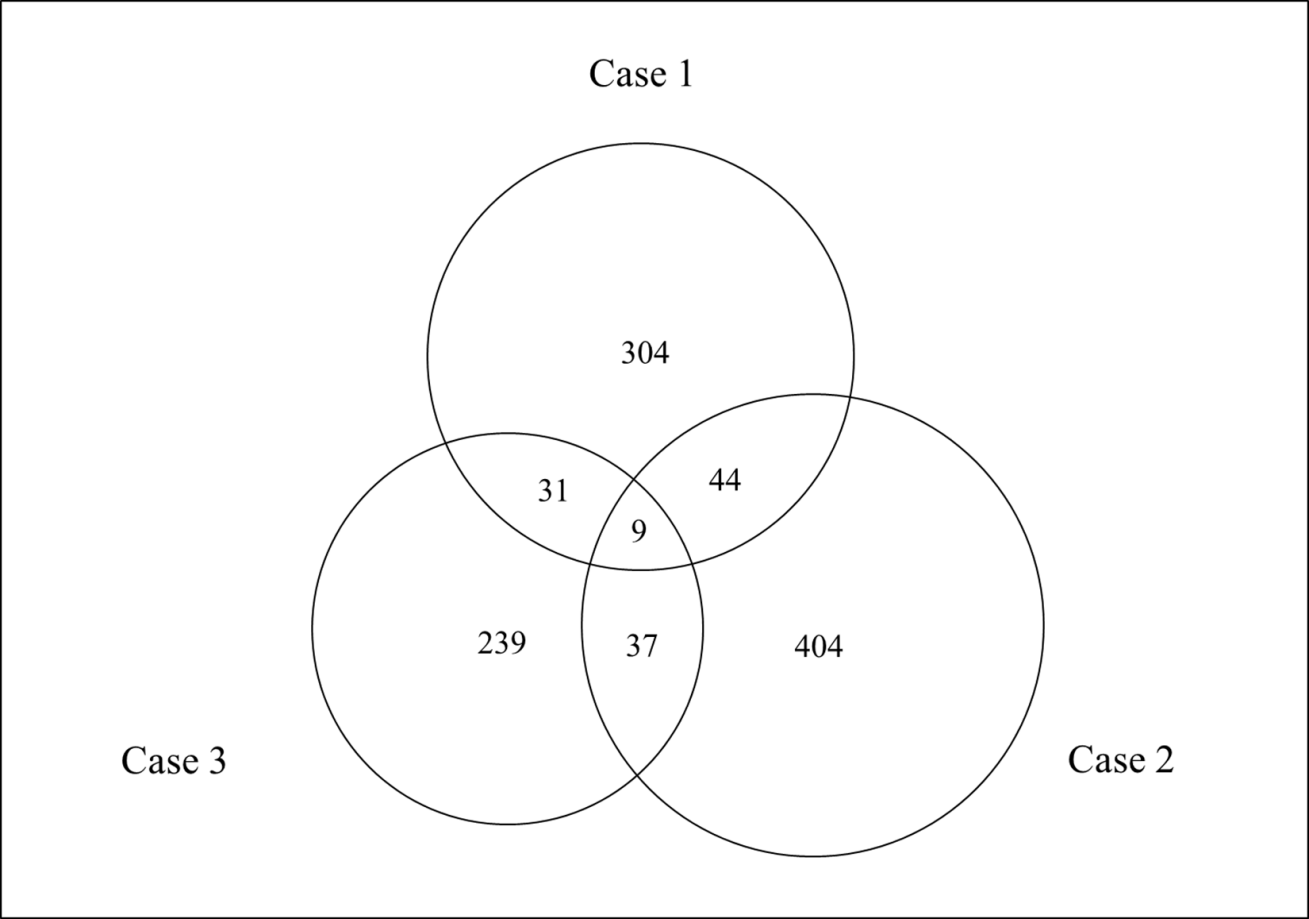
**Seromic analysis in sera from 3 patients with fulminant type 1 diabetes (FT1D) (Case 1, Case 2 and Case 3).** The Venn diagram shows the numbers of high signals (acute/sub-acute phase ratio>1.4) from 3 patients with FT1D. Nine antibodies which showed high signals in 3 patients with FT1D in the acute phase were detected by seromic analysis of 9418 antibodies.

In the seromic analysis of 9418 antibodies, we detected 9 antibodies which showed high signals from all 3 patients (Cases 1, 2 and 3) with FT1D especially in the acute phase (acute/sub-acute phase ratio >1.4) (Fig 1, Table 3 and S1 Table). Among the 9 candidate molecules, CD300e (CMRF35-like molecule 2) and LGALS3 (galectin-3) both serve important roles in immuno-regulation. In addition, the acute/sub-acute phase ratio of the CD300e signal was 10.02 in 1 subject (Case 3), representing the highest ratio among all antibodies. In the other two cases, the acute/sub-acute phase ratio of the CD300e signal was 1.49 (235th of all antibodies) in Case 1 and 2.51 (13th of all antibodies) in Case 2 (S1 Table). We therefore focused on these two antibodies, the anti-CD300e antibody and anti-LGALS3 antibody that have not been reported previously under any conditions.

**Table 3:** 9 candidate antibodies in seromic analysis.

CMRF35-like molecule (CD300E)
Galectin-3 (LGALS3)
Ribosomal protein S6 kinase, 70kDa, polypeptide 1 (RPS6KB1)
Bromodomain containing 3 (BRD3)
Beta-sarcoglycan (SGC B)
Disabled homolog 1 (Drosophila)(DAB1)
Chromosome 11 open reading frame 30 (C11orf30)
FERM domain containing 8 (FRMD8)
WAS/WASL-interacting (WIPF1)

### Anti-CD300e antibody measured by ELISA

**Fig 2:**
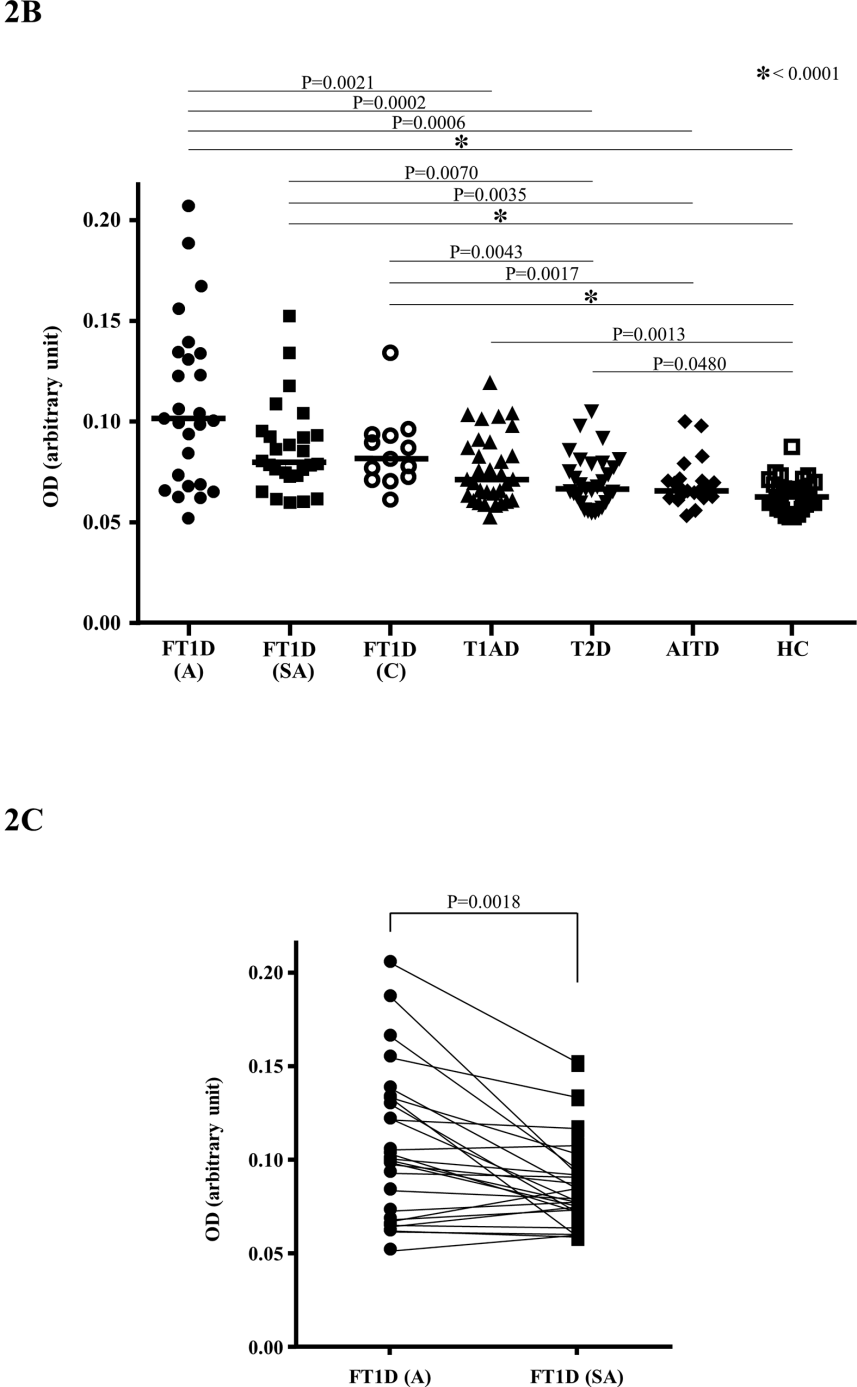
Titres of anti-CD300e antibody in sera, as determined by ELISA. A: Graph shows the titres of anti-CD300e antibody in sera from patients with fulminant type 1 diabetes in the acute phase (FT1D, A: n = 26), FT1D in the sub-acute phase (FT1D, SA: n = 26), FT1D in chronic phase (FT1D, C: n = 13), type 1A diabetes (T1AD: n = 32), type 2 diabetes (T2D: n = 30), autoimmune thyroid disease (AITD: n = 22) and healthy control subjects (HC: n = 31). The bars indicate the median. B: Graph shows comparison of the anti-CD300e antibody titres between FT1D patients in the acute phase (A) and those in the sub-acute phase (SA). The units are all OD (arbitrary unit).

The titres of the anti-CD300e antibody was 0.1000 (0.0515–0.2050) [median (range), arbitrary unit] in FT1D patients (acute phase, n = 26), 0.0784 (0.0583–0.1515) in FT1D patients (sub-acute phase, n = 26), 0.0828 (0.0624–0.1356) in FT1D patients (chronic phase, n = 13), 0.0721 (0.0534–0.1201) in T1AD patients, 0.0675 (0.0551–0.1056) in T2D patients, 0.0672 (0.0539–0.1007) in AITD patients and 0.0636 (0.0524–0.0877) in HC. A significantly higher titre of anti-CD300e antibody was detected in sera from FT1D patients in the acute phase (versus T1AD patients; P = 0.0021, versus T2D patients; P = 0.0002, versus AITD patients; P = 0.0006, versus HC; P<0.0001), and also in sera from FT1D patients both in the sub-acute phase (versus T2D patients; P = 0.0070, versus AITD patients; P = 0.0035, versus HC; P<0.0001) and that in the chronic phase (versus T2D patients; P = 0.0043, versus AITD patients; P = 0.0017, versus HC; P<0.0001) (Fig 2A). The titre of anti-CD300e antibody was higher in FT1D patients in the acute phase than those in the sub-acute phase (P = 0.0018, Wilcoxon signed-rank test). Significantly higher titre of anti-CD300e antibody was also detected in sera from both T1AD and T2D patients than that from HC (P = 0.0013, P = 0.0480, respectively) (Fig 2B).

There was no significant correlation between the titre of anti-CD300e antibody and age, gender, HbA1c level, and plasma glucose concentration in patients with FT1D, T1AD and T2D (data not shown). There was also no significant correlation between the titre of anti-CD300e antibody and that of GAD antibody or IA-2 antibody or fasting C-peptide level in patients with both FT1D and T1AD (data not shown).

### Specificity analysis of ELISAs by performing flow cytometry

**Fig 3:**
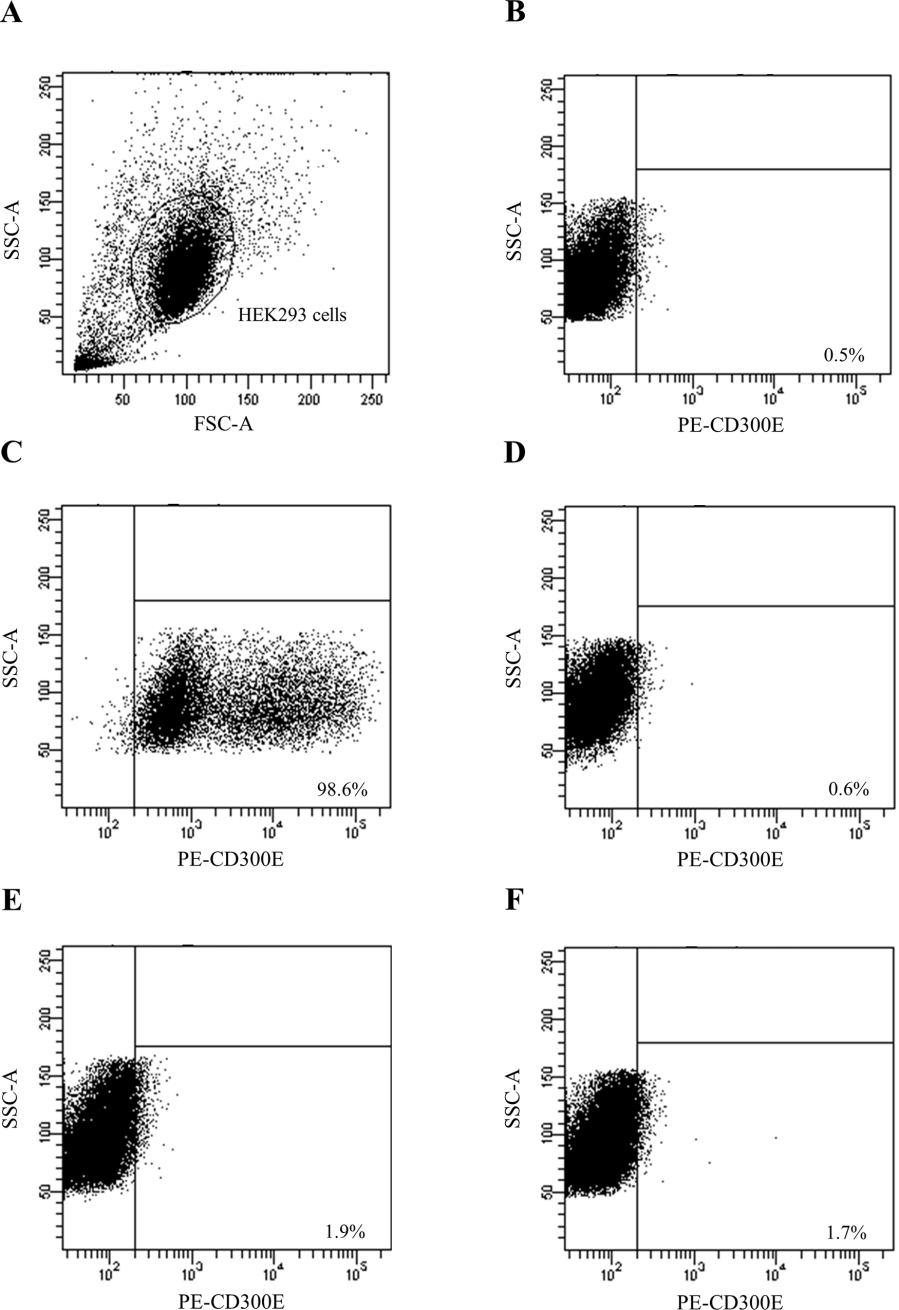
Specificity analysis of ELISAs by performing flow cytometry. Representative plots shows CD300e-transfected HEK293 cells (A) showing negative isotype control (B) or positive control by using PE-h CD300e antibody (C) or CD300e^+^ HEK293 cells by using sera from one HC (D) or CD300e^+^ HEK293 cells by using sera from 2 FT1D patients in the acute phase (E and F).

When we gated CD300e-transfected HEK293 cells (Fig 3A) and defined the target cell populations based on 0.5% of isotype controls (Fig 3B), 96.8% of all CD300e-transfected HEK293 cells were CD300e positive when PE-h CD300e antibody was used as a positive control (Fig 3C). CD300e^+^ HEK293 cells were confirmed by using sera from patients with acute-phase FT1D whose anti-CD300e antibody titres were the highest and the second highest by ELISA (Fig 3E and 3F). By contrast, few CD300e^+^ HEK293 cells were detected by using sera from HC (Fig 3D). When we investigated the correlation between the titre of anti-CD300e antibody by ELISA and the frequency of CD300e^+^ HEK293 cells by FACS in 17 subjects (10 FT1D in the acute phase and 7 HC), there was a significant positive correlation (r = 0.5221, P = 0.0316) (data not shown).

### Anti-LGALS3 antibody measured by ELISA

**Fig 4:**
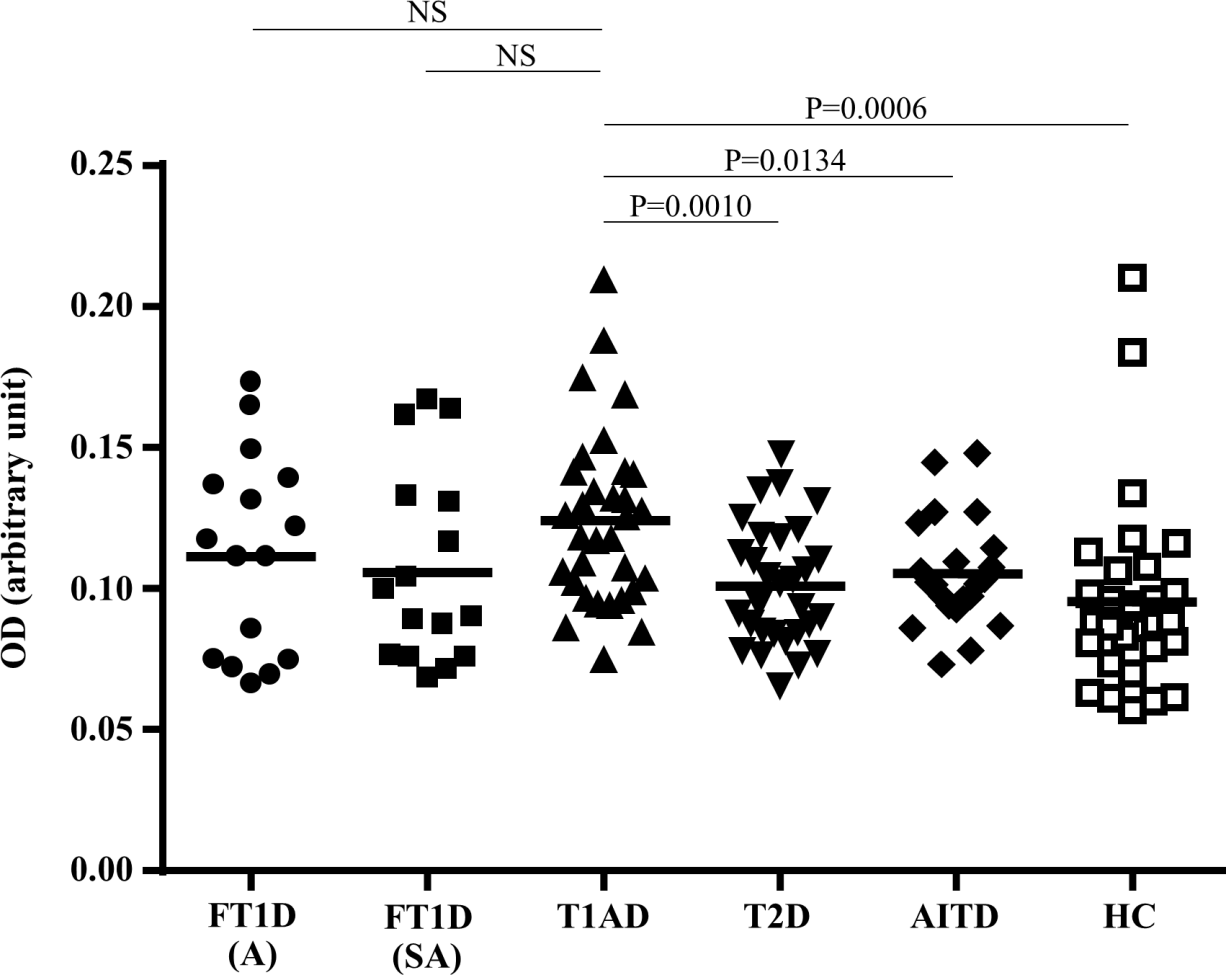
Titres of anti- LGALS3 antibody in sera, as determined by ELISA. Graph shows the titres of anti-LGALS3 antibody in sera from patients with fulminant type 1 diabetes in the acute phase (FT1D, A: n = 14), FT1D in the sub-acute phase (FT1D, SA: n = 14), type 1 diabetes (T1AD: n = 32), type 2 diabetes (T2D: n = 30), autoimmune thyroid disease (AITD: n = 22) and healthy control subjects (HC: n = 31). The bars indicate the mean.

The titre of anti-LGALS3 antibody was 0.1116 ± 0.0353 (mean ± SD, arbitrary unit) in FT1D patients (acute phase, n = 16), 0.1060 ± 0.0344 in FT1D patients (sub-acute phase, n = 16), 0.1245 ± 0.0310 in T1AD patients, 0.1011 ± 0.0213 in T2D patients, 0.1055 ± 0.0192 in AITD patients and 0.0956 ± 0.0329 in HC. Significantly higher titre of anti-LGALS3 antibody was detected in sera from T1AD patients (versus T2D patients; P = 0.0010, versus AITD patients; P = 0.0134, versus HC; P = 0.0006), but not in sera from FT1D patients in either the acute or sub-acute phase (Fig 4). The titre of anti-LGALS3 antibody in sera from patients with FT1D in the acute phase was not higher than those in patients with FT1D in the sub-acute phase, T2D, AITD or HC (Fig 4).

### ROC analysis of anti-CD300e antibody

**Fig 5:**
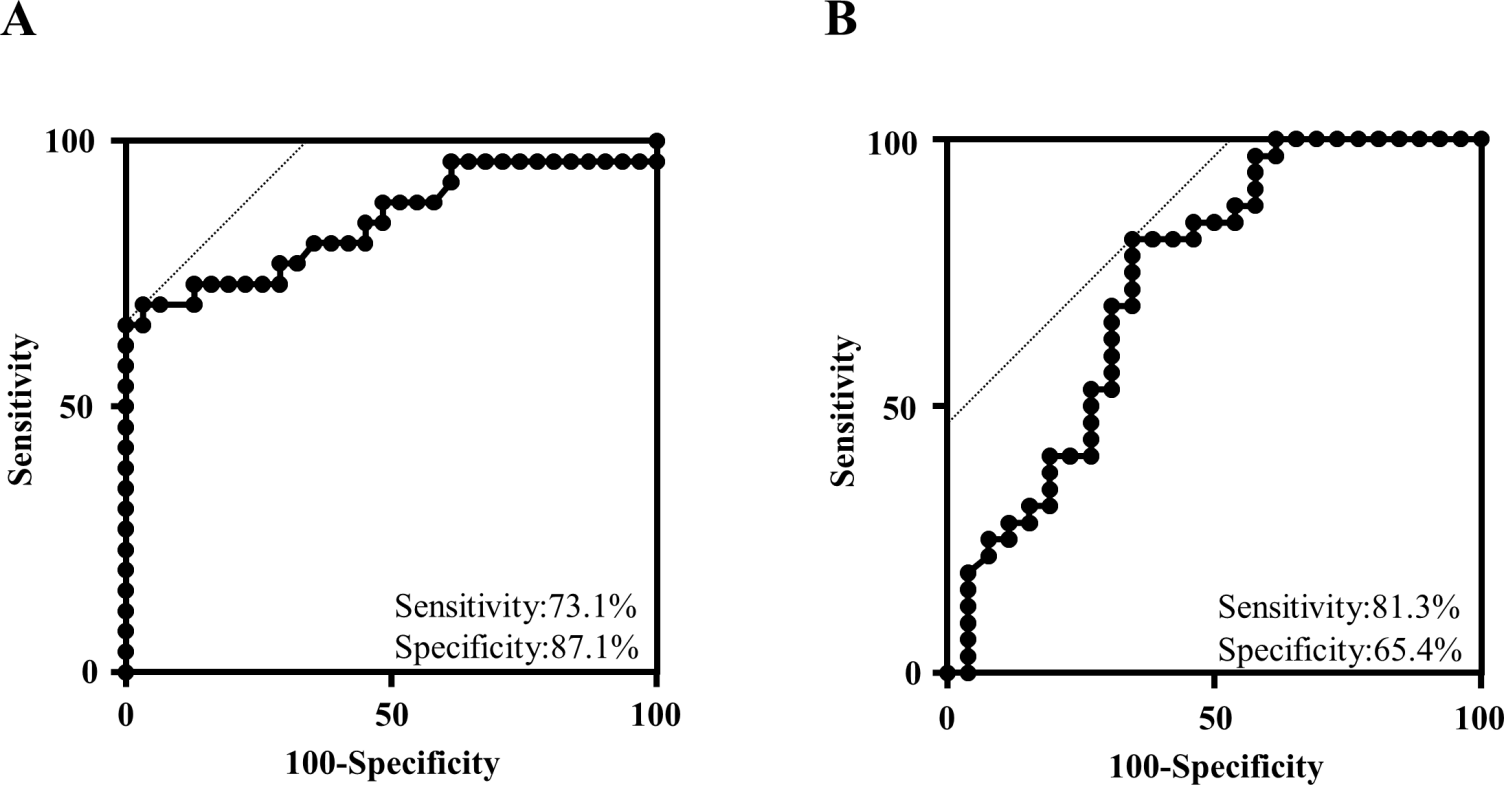
ROC analysis of anti-CD300e antibody. A: ROC analysis of patients with FT1D in the acute phase (n = 26) and HC (n = 30). B: ROC analysis of patients with FT1D in the acute phase (n = 26) and T1AD (n = 32).

When we carried out ROC analysis of anti-CD300e antibody in patients with FT1D in the acute phase (n = 26) and HC (n = 31), the area under the ROC curve was 0.849. The sensitivity and the specificity were 73.1% and 87.1%, respectively (Fig 5A). We also carried out ROC analysis of patients with FT1D in the acute phase (n = 26) and T1AD (n = 32). The area under the ROC curve was 0.733. The sensitivity and the specificity were 81.3% and 65.4%, respectively (Fig 5B). The cut-off value for a positive result in the ELISA analysis of anti-CD300e antibody on the basis of ROC analysis (Fig 5A) was 0.07208. Using this cut-off value, we found that the proportion of patients positive for anti-CD300e antibody in each of the groups in Fig 2A was 73.1% (FT1D in the acute phase), 73.1% (FT1D in the sub-acute phase), 84.6% (FT1D in the chronic phase), 53.1% (T1AD), 40.0% (T2D), 22.7% (AITD) and 12.9% (HC), respectively.

## Discussion

In current research, we detected a novel antibody to CD300e by applying seromic analysis which assessed the binding of IgG antibodies against a panel of more than 9000 human antigens in a small number of patients with FT1D. We then confirmed the presence of this antibody by conventional ELISA in a larger number of patients. The significance of our results is that 1) anti-CD300e antibody would be a novel biomarker for immediate diagnosis of FT1D and 2) CD300e would be a candidate of key molecule to elucidate the pathogenesis of FT1D.

We have clearly shown the high titre of anti-CD300e antibody in FT1D patients especially in acute phase. Moderate sensitivity and specificity were observed through ROC analysis of anti-CD300e antibody. Thus, anti-CD300e antibody would be a useful tool to diagnose FT1D. According to the diagnostic criteria by the Japan Diabetes Society, we must confirm 5 different kinds of data to diagnose FT1D. They are plasma glucose level, HbA1c concentration, serum (or urinary) C peptide level, serum (or urinary) ketone body level and the short history of diabetes [20, 21]. In addition to these 5 indices, anti-CD300e antibody might enable us to diagnose FT1D immediately in an emergency room or outpatient clinic. This is the first report that anti-CD300e antibody would be a biomarker of certain disease, although several reports have been published in which CD300 superfamily members have been used as biomarkers [22].

We confirmed the specificity of the ELISA assay by performing FACS analysis, although the titres of anti-CD300e antibody were generally low (the highest titre was < 0.20) in ELISA. There was a significant positive correlation between the titre of anti-CD300e antibody determined by ELISA and the frequency of CD300e^+^ HEK293 cells determined by FACS.

Anti-CD300e antibody might function as a key molecule in the development of FT1D via both innate immunity, by regulating the activity of macrophages/dendritic cells, and adaptive immunity, by modulating the activity of T cells. CD300e was originally cloned as a homologue of immune receptor expressed by myeloid cells 1 (IREM-1) and named as IREM-2 [23]. This molecule is a monomeric 32 kDa glycoprotein and possesses a single extracellular immunoglobulin domain. This immune receptor is known to be expressed by monocytes and myeloid dendritic cells (mDC) [24, 25]. The activation of CD300e by anti-CD300e antibody has been reported to trigger strong production of different pro-inflammatory cytokines, including TNF-α, IL-6 and IL-8/CXCL8 [25]. In FT1D, CD11+ dendritic cells are shown to be infiltrating to islets of the pancreas soon after disease onset [26]. The expression of CD300e has not been confirmed in islet-infiltrating cells in FT1D; however, most of CD11c+ cells are positive for CD300e [22], suggesting that islet-infiltrating CD11+ cells are also positive for CD300e and that pro-inflammatory cytokines such as TNF-alpha, released in response to anti-CD300e antibody, would directly damage beta cells in situ of the FT1D pancreas. In addition, the activation of CD300e by anti-CD300e antibody also increases expression of co-stimulatory molecules such as CD80 or CD86 in mDC, and the CD300e-stimulated mDC enhances the allo-reactive response of naive T cells [25]. These functions of CD300e might result in the proliferation of T cells via adaptive immune activation and lead to the destruction of pancreatic beta cells in the development of FT1D. In fact, T cell infiltration is also common in islets of the FT1D pancreas soon after disease onset [13].

It should be noted that the subclass of anti-CD300e antibody in the present study is IgG, not IgM. This finding suggests that immune reactions against CD300e had already started before the clinical onset of FT1D, at least a few weeks earlier, probably at the very first stage of the beta cell destruction in FT1D. In addition, significantly higher titre of anti-CD300e antibody was detected in sera from FT1D patients not only in the acute phase but also in the chronic phase, although the titre was lower compared with that in the acute phase, than that from patients with T2D, AITD or HC. This result indicates that the immune response to CD300e is a phenomenon specific to FT1D. Taken together, CD300e is suggested to play a role in the initiation of beta cell destruction, which is followed by the preclinical stage for a few weeks, resulting finally in overt diabetes in FT1D.

A higher titre of anti-LGALS3 antibody was detected in sera from patients with T1AD but not in those from patients with FT1D, either in the acute or sub-acute phase, compared patients with T2D or AITD or HC. In addition, the titre of anti-LGALS3 antibody did not differ between patients with T1AD and FT1D either in the acute or sub-acute phase. Galectin-3 activates macrophages and plays an important role in the proliferation of activated T cells [27–29]. Galectin-3-deficient mice produce lower levels of inflammatory cytokines such as IFN-gamma, TNF-alpha and IL-17 [30, 31]. Thus, the autoantibody to galectin-3, or anti-LGALS3 antibody, might play a role in the pathogenesis of T1D, especially in T1AD.

## Conclusion

We detected a novel antibody, anti-CD300e antibody of which the titre was high in sera from patients with FT1D especially in the acute phase. This antibody might be a diagnostic marker and provide new insight into the pathogenesis of FT1D.

## Supporting Information

S1 Table Antibodies for which the OD ratio of acute FT1D to sub-acute was >1.4 in seromic analysis. (DOCX)

## Abbreviations

FT1D: Fulminant type 1 diabetes
T1D: Type 1 diabetes
T1AD: Type 1A diabetes
T2D: Type 2 diabetes
AITD: Autoimmune thyroid disease
HC: Healthy control subject
ELISPOT: Enzyme-linked immunospot
mDC: Myeloid dendritic cell
ICA: Islet-cell antibodies
GAD: Glutamic acid decarboxylase
IA-2: Insulinoma associated antigen 2
ZnT8: Zinc transporter 8
