# Supplementary material for: Antibody to CMRF35-Like Molecule 2, CD300e A Novel Biomarker Detected in Patients with Fulminant Type 1 Diabetes
Source: PLoS One. 2016 Aug 11;11(8):e0160576. doi: 10.1371/journal.pone.0160576 (PMC4981355; doi:10.1371/journal.pone.0160576)
Supplement: S1 Table — (DOCX) [file pone.0160576.s001.docx]

**Table S1**

304 antibodies for which the OD ratio of acute FT1D to sub-acute was >1.4 in Case 1

splicing factor 3b, subunit 2, 145kDa (SF3B2)

phosphodiesterase 1A, calmodulin-dependent (PDE1A), transcript variant 1

mRNA similar to LOC149651 (cDNA clone MGC:39393 IMAGE:4862156), complete cds

Insulin gene enhancer protein ISL-1

Nucleoporin NUP53

family with sequence similarity 102, member A (FAM102A), transcript variant 2

BTB/POZ domain-containing protein KCTD18

replication factor C (activator 1) 4, 37kDa (RFC4), transcript variant 1

RAN binding protein 3 (RANBP3)

zinc finger CCCH-type, antiviral 1-like (ZC3HAV1L)

lectin, galactoside-binding, soluble, 7 (galectin 7) (LGALS7)

Cell division protein kinase 10

EPM2A (laforin) interacting protein 1 (EPM2AIP1)

p21(CDKN1A)-activated kinase 6 (PAK6)

NECAP endocytosis associated 1 (NECAP1)

chromosome 11 open reading frame 30 (C11orf30)

PCTAIRE protein kinase 1 (PCTK1), transcript variant 3

Purkinje cell protein 4 (PCP4)

p21(CDKN1A)-activated kinase 6 (PAK6)

origin recognition complex, subunit 6 like (yeast) (ORC6L)

forkhead box P3 (FOXP3)

Gamma-interferon-inducible protein Ifi-16

Acetyl-coenzyme A synthetase, cytoplasmic

signal-induced proliferation-associated 1 like 2 (SIPA1L2)

RNA-binding protein with multiple splicing

PRELI domain-containing protein 2

ankyrin repeat and MYND domain containing 1 (ANKMY1)

Ubiquitin-conjugating enzyme E2 E1

WAS/WASL-interacting protein family member 1

calcium/calmodulin-dependent protein kinase (CaM kinase) II alpha (CAMK2A)

Homeobox protein PKNOX2

pentatricopeptide repeat domain 2 (PTCD2)

Probable ATP-dependent RNA helicase DDX58

mitochondrial ribosomal protein L19 (MRPL19), nuclear gene encoding mitochondrial protein

cDNA clone IMAGE:5172086, partial cds

PCTAIRE protein kinase 1 (PCTK1)

amidohydrolase domain containing 1 (AMDHD1)

NA

potassium channel tetramerisation domain containing 18 (KCTD18)

lectin, galactoside-binding, soluble, 3 (LGALS3)

survival of motor neuron protein interacting protein 1 (SIP1), transcript variant alpha

nuclear import 7 homolog (S. cerevisiae) (NIP7)

tau tubulin kinase 2 (TTBK2)

Rho guanine nucleotide exchange factor (GEF) 5 (ARHGEF5)

PRP4 pre-mRNA processing factor 4 homolog (yeast) (PRPF4)

lamin A/C (LMNA), transcript variant 2

ADP-ribosylation factor-like 4C (ARL4C)

H2A histone family, member Y (H2AFY)

guanosine monophosphate reductase (GMPR)

ribosomal protein S6 kinase, 70kDa, polypeptide 1 (RPS6KB1)

protein kinase C and casein kinase substrate in neurons 1 (PACSIN1)

lymphotoxin beta (TNF superfamily, member 3) (LTB), transcript variant 2

cyclin-dependent kinase inhibitor 1B (p27, Kip1) (CDKN1B)

chromobox homolog 3 (HP1 gamma homolog, Drosophila) (CBX3)

WD repeat domain 61 (WDR61)

septin 11 (SEPT11)

lectin, galactoside-binding, soluble, 1 (galectin 1) (LGALS1)

interleukin 20 (IL20)

protein phosphatase 1, regulatory (inhibitor) subunit 8 (PPP1R8), transcript variant 3

peroxisomal biogenesis factor 19 (PEX19)

inhibitor of growth family, member 5 (ING5)

Adipophilin

potassium channel tetramerisation domain containing 14 (KCTD14)

actin binding LIM protein 1 (ABLIM1)

uroporphyrinogen decarboxylase (UROD)

cancer/testis antigen family 45, member A1 (CT45A1), mRNA.

matrix metallopeptidase 8 (neutrophil collagenase) (MMP8)

RAB33A, member RAS oncogene family (RAB33A)

interferon regulatory factor 9 (ISGF3G)

N-glycanase 1 (NGLY1)

phospholipase C-like 2 (PLCL2)

Ras-related protein Rab-7a

Protein Dos

RAN binding protein 5 (RANBP5)

endoplasmic reticulum protein 29 (ERP29), transcript variant 1

Syntaxin-binding protein 1

fibronectin 1 (FN1)

Serine/threonine-protein kinase 6

ubiquitin-conjugating enzyme E2B (RAD6 homolog) (UBE2B)

isopentenyl-diphosphate delta isomerase 2 (IDI2)

CDC42 effector protein (Rho GTPase binding) 2 (CDC42EP2)

Syntaxin-binding protein 2

Putative ATP-dependent Clp protease proteolytic subunit, mitochondrial

chromosome 1 open reading frame 63 (C1orf63)

synaptotagmin VI (SYT6)

apolipoprotein B mRNA editing enzyme, catalytic polypeptide-like 3F (APOBEC3F), transcript variant 2, mRNA.

deoxynucleotidyltransferase, terminal (DNTT), transcript variant 1

NADH dehydrogenase (ubiquinone) flavoprotein 3, 10kDa (NDUFV3)

RecQ protein-like (DNA helicase Q1-like) (RECQL)

T-box 22 (TBX22), transcript variant 2

septin 10 (SEPT10), transcript variant 1

platelet-activating factor acetylhydrolase, isoform Ib, alpha subunit 45kDa (PAFAH1B1)

ATP-binding cassette, sub-family F (GCN20), member 2 (ABCF2), nuclear gene encoding mitochondrial protein, transcript variant 1

PREDICTED: Homo sapiens hypothetical protein LOC284276 (LOC284276)

Homeobox protein Hox-B6

chromosome 3 open reading frame 36 (C3orf36)

protein kinase C and casein kinase substrate in neurons 2 (PACSIN2)

Microtubule-associated protein 9

family with sequence similarity 90, member A1 (FAM90A1)

Zinc finger protein 165

p21 (CDKN1A)-activated kinase 2 (PAK2)

Mediator of RNA polymerase II transcription subunit 29

hsp70-interacting protein (HSPBP1)

Myosin light chain 2, lymphocyte-specific

retinitis pigmentosa 2 (X-linked recessive) (RP2)

phosphodiesterase 4D, cAMP-specific (phosphodiesterase E3 dunce homolog, Drosophila) (PDE4D)

HIRA interacting protein 3 (HIRIP3)

cyclin B2 (CCNB2)

RASD family, member 2 (RASD2)

mediator of RNA polymerase II transcription, subunit 8 homolog (S. cerevisiae) (MED8), transcript variant 4

piccolo (presynaptic cytomatrix protein) (PCLO)

syntaxin binding protein 5 (tomosyn) (STXBP5)

leukocyte receptor cluster (LRC) member 1 (LENG1)

septin 4 (SEPT4), transcript variant 3

Serine/threonine-protein kinase 6

G1 to S phase transition 2 (GSPT2)

Methyl-CpG-binding domain protein 3

germ cell-less homolog 1 (Drosophila)-like (GMCL1L)

FERM domain containing 8 (FRMD8)

Beta-sarcoglycan

Protein tyrosine kinase 2 beta

calcium channel, voltage-dependent, beta 1 subunit (CACNB1), transcript variant 1

chromosome X open reading frame 56 (CXorf56)

C-X-C motif chemokine 11

phosphoglycerate mutase 1 (brain) (PGAM1)

proopiomelanocortin (adrenocorticotropin/ beta-lipotropin/ alpha-melanocyte stimulating hormone/ beta-melanocyte stimulating hormone/ beta-endorphin) (POMC), transcript variant 2

RNA-binding protein 40

DnaJ (Hsp40) homolog, subfamily B, member 2 (DNAJB2)

RAB8B, member RAS oncogene family (RAB8B)

nucleoporin 50kDa (NUP50), transcript variant 3

sciellin (SCEL)

Uncharacterized protein C8orf41

JTV1 gene (JTV1)

crumbs homolog 3 (Drosophila) (CRB3), transcript variant 2

MAP/microtubule affinity-regulating kinase 2 (MARK2), transcript variant 3

Protein Dr1

dynein, light chain, roadblock-type 2 (DYNLRB2)

transglutaminase 2 (C polypeptide, protein-glutamine-gamma-glutamyltransferase) (TGM2)

zinc finger, ZZ-type containing 3 (ZZZ3)

Treacher Collins-Franceschetti syndrome 1 (TCOF1), transcript variant 3

cancer/testis antigen CT45-3 (CT45-3)

Coiled-coil domain-containing protein 46

Regulator of G-protein signaling 8

Leptin receptor gene-related protein

alcohol dehydrogenase 5 (class III), chi polypeptide (ADH5)

myosin, light chain 2, regulatory, cardiac, slow (MYL2)

nicalin homolog (zebrafish) (NCLN)

2prime,5prime-phosphodiesterase 12

Protein asteroid homolog 1

Alpha-ketoglutarate-dependent dioxygenase alkB homolog 3

NADH dehydrogenase (ubiquinone) Fe-S protein 4, 18kDa (NADH-coenzyme Q reductase) (NDUFS4)

Alpha-catulin

signal sequence receptor, gamma (translocon-associated protein gamma) (SSR3)

TBC1 domain family, member 10C (TBC1D10C)

chromosome 12 open reading frame 26 (C12orf26)

Proteasome assembly chaperone 2

tumor protein D52-like 3 (TPD52L3)

NIMA (never in mitosis gene a)- related kinase 7 (NEK7)

protein phosphatase 1, regulatory (inhibitor) subunit 14A (PPP1R14A)

ISL LIM homeobox 1 (ISL1)

SET binding protein 1 (SETBP1)

JTV1 gene (JTV1)

signal peptidase complex subunit 1 homolog (S. cerevisiae) (SPCS1)

zinc finger protein 688 (ZNF688), transcript variant 1

coiled-coil domain containing 72 (CCDC72)

FLJ46266 protein (FLJ46266), mRNA.

secretogranin III (SCG3)

N-acetyltransferase 6 (NAT6)

ribosomal protein S6 kinase, 70kDa, polypeptide 1 (RPS6KB1)

Pumilio domain-containing protein KIAA0020

ubiquitin-conjugating enzyme E2N (UBC13 homolog, yeast) (UBE2N)

family with sequence similarity 44, member A (FAM44A)

deafness, autosomal dominant 5 (DFNA5)

Cyclin-Y

ghrelin/obestatin preprohormone (GHRL)

jagged 1 (Alagille syndrome) (JAG1)

TCR gamma alternate reading frame protein (TARP), nuclear gene encoding mitochondrial protein, transcript variant 1

chromosome 11 open reading frame 52 (C11orf52)

alveolar soft part sarcoma chromosome region, candidate 1 (ASPSCR1)

fibronectin type III and SPRY domain containing 1-like (FSD1L), transcript variant 1

Voltage-dependent L-type calcium channel subunit beta-1

chromosome 19 open reading frame 43 (C19orf43)

bromodomain containing 3 (BRD3)

NADH dehydrogenase [ubiquinone] iron-sulfur protein 4, mitochondrial

Ral GEF with PH domain and SH3 binding motif 1 (RALGPS1)

PRP4 pre-mRNA processing factor 4 homolog (yeast) (PRPF4)

protein tyrosine phosphatase domain containing 1, mRNA (cDNA clone MGC:70358 IMAGE:5539182), complete cds

AF4/FMR2 family, member 4 (AFF4)

keratin associated protein 3-3 (KRTAP3-3)

nuclear transport factor 2-like export factor 2 (NXT2)

DDI1, DNA-damage inducible 1, homolog 2 (S. cerevisiae) (DDI2)

DIM1 dimethyladenosine transferase 1-like (S. cerevisiae) (DIMT1L)

processing of precursor 5, ribonuclease P/MRP subunit (S. cerevisiae) (POP5), transcript variant 1

paxillin (PXN)

translin-associated factor X interacting protein 1 (TSNAXIP1)

spermatogenesis associated, serine-rich 2 (SPATS2)

C1q and tumor necrosis factor related protein 9 (C1QTNF9)

RAB10, member RAS oncogene family (RAB10)

polymerase (RNA) II (DNA directed) polypeptide K, 7.0kDa (POLR2K)

RNA binding motif protein 41 (RBM41)

Glutaminyl-tRNA synthetase

TOM1-like protein 2

PREDICTED: Homo sapiens similar to CG14853-PB (LOC285141)

vacuolar protein sorting 41 homolog (S. cerevisiae) (VPS41)

Acyl-CoA-binding domain-containing protein 4

sorting nexin 7 (SNX7)

Differentially expressed in FDCP 6 homolog

Fanconi anemia, complementation group M (FANCM)

disabled homolog 1 (Drosophila) (DAB1)

Nuclear nucleic acid-binding protein C1D

mitochondrial methionyl-tRNA formyltransferase (MTFMT)

Protein midA homolog, mitochondrial

ubiquitin protein ligase E3A (human papilloma virus E6-associated protein, Angelman syndrome) (UBE3A)

peptidyl arginine deiminase, type IV (PADI4)

Tetratricopeptide repeat protein 15

leucine rich repeat containing 1 (LRRC1)

fibronectin type III domain containing 4 (FNDC4)

Serine/threonine-protein kinase Chk2

apolipoprotein B mRNA editing enzyme, catalytic polypeptide-like 3G (APOBEC3G)

L-asparaginase

Phosphoglycerate mutase 1

lin-7 homolog A (C. elegans) (LIN7A)

SYF2 homolog, RNA splicing factor (S. cerevisiae) (SYF2), transcript variant 1

integral membrane protein 2C (ITM2C)

chromosome 1 open reading frame 62 (C1orf62)

Interferon-inducible double stranded RNA-dependent protein kinase activator A

Rho GTPase-activating protein 12

Homeobox protein Meis1

RasGEF domain family, member 1B (RASGEF1B)

PREDICTED: Homo sapiens hypothetical LOC400523 (LOC400523)

regulator of G-protein signaling 8 (RGS8), transcript variant 1

chromosome 17 open reading frame 77 (C17orf77)

TBC1 domain family member 13

CMRF35-like molecule 2

DNA topoisomerase 3-beta-1

cartilage acidic protein 1 (CRTAC1)

chromosome 5 open reading frame 20 (C5orf20)

T-box transcription factor TBX20

chromosome 6 open reading frame 134 (C6orf134)

paraneoplastic antigen MA2 (PNMA2)

Coiled-coil domain-containing protein C1orf110

cell division cycle 25 homolog C (S. pombe) (CDC25C), transcript variant 1

ADP-ribosylation factor-like 4A (ARL4A), transcript variant 1

dynein, axonemal, light chain 4 (DNAL4)

exonuclease 3'-5' domain-like 2 (EXDL2)

Melanoma-associated antigen 2

centrosomal protein 57kDa (CEP57)

forkhead box A3 (FOXA3)

nucleoporin 50kDa (NUP50), transcript variant 2

Glutaminyl-tRNA synthetase

centaurin, alpha 2 (CENTA2)

PREDICTED: Homo sapiens hypothetical LOC387974 (LOC387974)

sorbitol dehydrogenase (SORD)

Galectin-3

aspartoacylase (aminocyclase) 3 (ACY3)

Phosphatidylinositol 4-kinase beta

H2A histone family, member Y2 (H2AFY2)

lectin, galactoside-binding, soluble, 8 (galectin 8) (LGALS8)

melanoma antigen family B, 1 (MAGEB1), transcript variant 1

thioredoxin domain containing 9 (TXNDC9)

ADP-ribosylation factor GTPase activating protein 3 (ARFGAP3)

replication protein A4, 34kDa (RPA4)

Serine/threonine-protein kinase PCTAIRE-2

zeta-chain (TCR) associated protein kinase 70kDa (ZAP70)

Proline-rich protein 16

double homeobox, 3 (DUX3)

ubiquitin D (UBD)

erbb2 interacting protein (ERBB2IP), transcript variant 2

protein kinase C, zeta (PRKCZ)

gamma-aminobutyric acid (GABA) A receptor, alpha 4 (GABRA4)

hypothetical protein MGC31957 (MGC31957)

chromosome 10 open reading frame 63 (C10orf63)

TAR DNA binding protein (TARDBP)

regulatory factor X, 5 (influences HLA class II expression) (RFX5), transcript variant 1

DKFZP434K028 protein (DKFZP434K028)

MAP kinase-activated protein kinase 3

stomatin (EPB72)-like 1 (STOML1)

sialic acid binding Ig-like lectin 6 (SIGLEC6)

melanoma antigen family B, 2 (MAGEB2)

PDS5, regulator of cohesion maintenance, homolog A (S. cerevisiae) (SCC-112)

kinesin family member 6 (KIF6)

DnaJ (Hsp40) homolog, subfamily B, member 6 (DNAJB6)

trafficking protein particle complex 1 (TRAPPC1)

regulator of G-protein signaling 13 (RGS13), transcript variant 1

Uncharacterized protein C17orf85

BCL2-like 13 (apoptosis facilitator) (BCL2L13)

MOB1, Mps One Binder kinase activator-like 2A (yeast) (MOBKL2A)

chromatin modifying protein 2B (CHMP2B)

Rap guanine nucleotide exchange factor (GEF) 4 (RAPGEF4)

hexamthylene bis-acetamide inducible 2 (HEXIM2)

polo-like kinase 3 (Drosophila) (PLK3)

RAS-like, family 11, member B (RASL11B)

Zinc fingers and homeoboxes protein 1

F-box protein 21 (FBXO21)

Nuclear pore complex protein Nup133

SLAIN motif family, member 2 (SLAIN2)

tyrosine 3-monooxygenase/tryptophan 5-monooxygenase activation protein, beta polypeptide, mRNA (cDNA clone IMAGE:4292790), complete cds.

Metastasis-associated protein MTA1

Cell division protein kinase 10

chromosome 9 open reading frame 41 (C9orf41)

Dual specificity mitogen-activated protein kinase kinase 5

tyrosine 3-monooxygenase/tryptophan 5-monooxygenase activation protein, zeta polypeptide (YWHAZ), transcript variant 1

Ephrin receptor A5 (EPHA5), transcript variant 1

Pyruvate kinase isozymes R/L

404 antibodies for which the OD ratio of acute FT1D to sub-acute was >1.4 in Case 2

palmitoyl-protein thioesterase 2 (PPT2), transcript variant 1

Metalloproteinase inhibitor 1

Wiskott-Aldrich syndrome-like (WASL)

Usher syndrome 1C (autosomal recessive, severe) (USH1C)

chromosome 11 open reading frame 30 (C11orf30)

uridine monophosphate synthetase (UMPS), mRNA.

Paladin

S100 calcium binding protein A12 (S100A12)

TSC22 domain family, member 1 (TSC22D1), transcript variant 2

eukaryotic translation initiation factor 3, subunit G (EIF3S4)

Protein FAM46B

CMRF35-like molecule 2

Beta-sarcoglycan

lectin, galactoside-binding, soluble, 1 (galectin 1) (LGALS1)

TBC1 domain family, member 10C (TBC1D10C)

Uncharacterized protein C12orf65

cDNA clone MGC:23888 IMAGE:4704496, complete cds

interferon regulatory factor 6 (IRF6)

Condensin complex subunit 3

PREDICTED: Homo sapiens hypothetical protein LOC284276 (LOC284276)

lectin, galactoside-binding, soluble, 8 (galectin 8) (LGALS8)

adaptor-related protein complex 3, mu 1 subunit (AP3M1), transcript variant 2

p21(CDKN1A)-activated kinase 6 (PAK6)

quinolinate phosphoribosyltransferase (nicotinate-nucleotide pyrophosphorylase (carboxylating)) (QPRT)

ubiquitin domain containing 2 (UBTD2)

v-ets erythroblastosis virus E26 oncogene homolog (avian) (ERG), transcript variant 2

coiled-coil-helix-coiled-coil-helix domain containing 2 (CHCHD2)

F-box protein 21 (FBXO21)

cardiotrophin-like cytokine factor 1 (CLCF1)

nerve growth factor receptor (TNFRSF16) associated protein 1 (NGFRAP1), transcript variant 1

thiopurine S-methyltransferase (TPMT)

Hypermethylated in cancer 2 protein

UPF0556 protein C19orf10

ribosomal protein S6 kinase, 70kDa, polypeptide 1 (RPS6KB1)

Homeobox protein TGIF2LX

neurotensin (NTS)

cache domain containing 1 (CACHD1)

pleckstrin homology-like domain, family A, member 1 (PHLDA1)

nucleoporin like 1 (NUPL1), transcript variant 1

peroxisomal membrane protein 4, 24kDa (PXMP4), transcript variant 2

peroxisome proliferator-activated receptor gamma (PPARG)

protein kinase C and casein kinase substrate in neurons 2 (PACSIN2)

ADP-ribosylation factor-like protein 6-interacting protein 4

vacuolar protein sorting 26 homolog A (S. pombe) (VPS26A), transcript variant 1

H2A histone family, member Z (H2AFZ)

armadillo repeat containing 8 (ARMC8)

prefoldin subunit 5 (PFDN5), transcript variant 1

Pentatricopeptide repeat-containing protein 3, mitochondrial

nuclear factor I/A (NFIA)

general transcription factor II, i (GTF2I), transcript variant 4

Endophilin-B2

tRNA-specific adenosine deaminase-like protein 3

CDGSH iron sulfur domain 1 (CISD1)

MAP kinase-activated protein kinase 2

forkhead box P4 (FOXP4)

Tumor protein D52

lectin, galactoside-binding, soluble, 8 (galectin 8) (LGALS8)

tryptophanyl-tRNA synthetase (WARS), transcript variant 3

serine dehydratase (SDS)

ribosomal protein S6 kinase, 70kDa, polypeptide 1 (RPS6KB1)

Interleukin-1 receptor-associated kinase 1-binding protein 1

CD46 molecule, complement regulatory protein (CD46), transcript variant d

cytoplasmic FMR1 interacting protein 2 (CYFIP2), transcript variant 3

vitamin D (1,25- dihydroxyvitamin D3) receptor (VDR), transcript variant 1

RAS (RAD and GEM)-like GTP-binding 1 (REM1)

mahogunin, ring finger 1 (MGRN1)

Angiogenic factor with G patch and FHA domains 1

purinergic receptor P2Y, G-protein coupled, 2 (P2RY2)

Homeobox protein TGIF2LX

Transketolase

H2A histone family, member V (H2AFV), transcript variant 4

hypothetical LOC554203 (LOC554203)

Putative protein phosphatase inhibitor 2-like protein 3

NA

uroplakin 3A (UPK3A)

ADP-ribosylation factor-like 4A (ARL4A), transcript variant 1

ethylmalonic encephalopathy 1 (ETHE1)

chromosome 16 open reading frame 74 (C16orf74)

MRG-binding protein

E2F transcription factor 4, p107/p130-binding (E2F4)

EPM2A (laforin) interacting protein 1 (EPM2AIP1)

spondin 2, extracellular matrix protein (SPON2)

NECAP endocytosis associated 1 (NECAP1)

centrosome and spindle pole associated protein 1 (CSPP1), transcript variant 2

leukocyte receptor cluster (LRC) member 1 (LENG1)

oligonucleotide/oligosaccharide-binding fold containing 1 (OBFC1)

cell division cycle 2, G1 to S and G2 to M (CDC2), transcript variant 1

BMX non-receptor tyrosine kinase (BMX), transcript variant 2

Galectin-3

BolA-like protein 3

phosphate cytidylyltransferase 2, ethanolamine (PCYT2)

RNA binding motif protein 41 (RBM41)

cytochrome c oxidase subunit VIa polypeptide 1 (COX6A1), nuclear gene encoding mitochondrial protein

TBC1 domain family, member 22A (TBC1D22A)

v-abl Abelson murine leukemia viral oncogene homolog 1 (ABL1), transcript variant a; see catalog number for detailed information on wild-type or point mutant status

GRIP and coiled-coil domain-containing protein 1

lectin, galactoside-binding, soluble, 3 (LGALS3)

Rap guanine nucleotide exchange factor (GEF) 4 (RAPGEF4)

Mediator of RNA polymerase II transcription subunit 29

RNA binding motif protein 39 (RBM39), transcript variant 3

tubulin polymerization promoting protein (TPPP)

small nuclear ribonucleoprotein polypeptide B'' (SNRPB2), transcript variant 1

kelch-like 29 (Drosophila) (KLHL29)

A.T hook DNA-binding motif-containing protein 1

similar to envelope protein (LOC113386)

regulator of calcineurin 1 (RCAN1), transcript variant 2

spermine oxidase (SMOX), transcript variant 2

JmjC domain-containing histone demethylation protein 3B

DnaJ (Hsp40) homolog, subfamily A, member 4 (DNAJA4)

amelogenin (amelogenesis imperfecta 1, X-linked) (AMELX), transcript variant 1

zinc finger protein 740 (ZNF740)

poly (ADP-ribose) polymerase family, member 11 (PARP11)

regulator of calcineurin 1 (RCAN1), transcript variant 1

Uncharacterized protein C15orf39

ADP-ribosylation factor-like 8B (ARL8B)

RNA-binding protein 39

N-acylsphingosine amidohydrolase (acid ceramidase)-like (ASAHL), transcript variant 1

NUAK family, SNF1-like kinase, 2 (NUAK2)

mitogen-activated protein kinase 7 (MAPK7), transcript variant 3

ATP synthase, H+ transporting, mitochondrial F0 complex, subunit d (ATP5H), nuclear gene encoding mitochondrial protein, transcript variant 1

tektin 4 (TEKT4)

phosphoinositide-3-kinase, catalytic, delta polypeptide and phosphoinositide-3-kinase, regulatory subunit 1 (alpha): PIK3CD and PIK3R1 sequences are seperated by -- (in the protein list file).

chromosome 20 open reading frame 39 (C20orf39)

lectin, galactoside-binding, soluble, 7 (galectin 7) (LGALS7)

scinderin (SCIN)

WAS/WASL-interacting protein family member 1

chromosome 1 open reading frame 174 (C1orf174)

Kanadaptin

Coiled-coil domain-containing protein 126

zinc finger protein 533 (ZNF533)

Tyrosine-protein kinase ABL2

prefoldin subunit 2 (PFDN2)

TruB pseudouridine (psi) synthase homolog 1 (E. coli) (TRUB1)

chromosome 10 open reading frame 59 (C10orf59)

PH domain-containing protein C10orf81

armadillo repeat containing 7 (ARMC7)

chromosome 2 open reading frame 47 (C2orf47)

Kruppel-like factor 8 (KLF8)

small proline-rich protein 4 (SPRR4)

DnaJ (Hsp40) homolog, subfamily C, member 12 (DNAJC12), transcript variant 2

Alpha-1,3-mannosyl-glycoprotein 4-beta-N-acetylglucosaminyltransferase B

dysbindin (dystrobrevin binding protein 1) domain containing 2 (DBNDD2)

enoyl Coenzyme A hydratase domain containing 1 (ECHDC1)

SYS1 Golgi-localized integral membrane protein homolog (S. cerevisiae) (SYS1), transcript variant 1

NA

calcium homeostasis endoplasmic reticulum protein (CHERP)

phosphodiesterase 4D, cAMP-specific (phosphodiesterase E3 dunce homolog, Drosophila) (PDE4D)

glycine-N-acyltransferase-like 1 (GLYATL1)

RING finger protein 135

2,4-dienoyl CoA reductase 2, peroxisomal (DECR2)

SH3 domain and tetratricopeptide repeats 1 (SH3TC1)

Tigger transposable element-derived protein 1

activating transcription factor 6 (ATF6)

Spindlin-2B

family with sequence similarity 131, member C (FAM131C)

amyloid beta (A4) precursor protein-binding, family A, member 2 binding protein (APBA2BP)

Histone H2B type 1-H

coiled-coil domain containing 55 (CCDC55), transcript variant 1

Receptor expression-enhancing protein 1

ribosomal protein S19 (RPS19)

pleckstrin and Sec7 domain containing 3 (PSD3), transcript variant 2

sorbitol dehydrogenase (SORD)

erythrocyte membrane protein band 4.9 (dematin) (EPB49)

kelch domain containing 4 (KLHDC4)

DNA-directed RNA polymerase III subunit RPC9

bridging integrator 1 (BIN1)

Peptidylprolyl isomerase-like 5

muted homolog (mouse) (MUTED)

exocyst complex component 7 (EXOC7)

zinc finger, AN1-type domain 5 (ZFAND5), transcript variant c

chromosome 12 open reading frame 31 (C12orf31)

myotubularin related protein 14 (MTMR14)

Vacuolar protein sorting-associated protein 26B

Brain and acute leukemia cytoplasmic protein

3-hydroxybutyrate dehydrogenase, type 2 (BDH2)

WD repeat-containing protein mio

peptidylprolyl isomerase G (cyclophilin G) (PPIG)

SRY (sex determining region Y)-box 5 (SOX5)

chromosome 14 open reading frame 80 (C14orf80)

immunoglobulin lambda-like polypeptide 1 (IGLL1), transcript variant 1

heat shock 60kDa protein 1 (chaperonin) (HSPD1), nuclear gene encoding mitochondrial protein, transcript variant 1

BMX non-receptor tyrosine kinase (BMX), transcript variant 2

cell division cycle 25B (CDC25B), transcript variant 5

sperm flagellar 1 (SPEF1)

dynein, light chain, roadblock-type 2 (DYNLRB2)

UBX domain containing 1 (UBXD1)

feline sarcoma oncogene (FES)

Disks large homolog 3

mediator complex subunit 29 (MED29)

adrenergic, beta-2-, receptor, surface (ADRB2)

Polycomb protein EZH2

vacuolar protein sorting 16 homolog (S. cerevisiae) (VPS16), transcript variant 2

ankyrin repeat and MYND domain containing 1 (ANKMY1)

thyroid hormone receptor, alpha (erythroblastic leukemia viral (v-erb-a) oncogene homolog, avian) (THRA), transcript variant 1

GTF2I repeat domain containing 2 (GTF2IRD2)

mitogen-activated protein kinase kinase kinase 7 (MAP3K7), transcript variant D, mRNA.

protein kinase C, alpha (PRKCA); see catalog number for detailed information on wild-type or point mutant status

DnaJ homolog subfamily A member 3, mitochondrial

SH3 domain binding glutamic acid-rich protein like 2 (SH3BGRL2)

FERM domain containing 8 (FRMD8)

regulator of calcineurin 1 (RCAN1), transcript variant 3

taspase, threonine aspartase, 1 (TASP1)

ribosomal protein L39-like (RPL39L)

Glycogen synthase kinase-3 beta

immunoglobulin lambda variable 2-14 (IGLV2-14)

similar to pleckstrin homology domain containing, family M (with RUN domain) member 1; adapter protein 162, mRNA, complete cds.

zinc finger protein 684 (ZNF684)

ATP-binding cassette sub-family D member 4

bridging integrator 1 (BIN1), transcript variant 6

ariadne homolog 2 (Drosophila) (ARIH2)

mitogen-activated protein kinase kinase kinase 11 (MAP3K11)

myozenin 2 (MYOZ2)

CPX chromosome region, candidate 1 (CPXCR1)

family with sequence similarity 39, member B (FAM39B)

PREDICTED: Homo sapiens hypothetical protein LOC147646 (LOC147646), mRNA.

guanidinoacetate N-methyltransferase (GAMT)

single stranded DNA binding protein 3 (SSBP3), transcript variant 1

Ubiquitin carboxyl-terminal hydrolase 24

SPANX family, member B1 (SPANXB1)

Purine nucleoside phosphorylase

zinc finger, MYND domain containing 11 (ZMYND11)

SWI/SNF-related matrix-associated actin-dependent regulator of chromatin subfamily A member 5

nudE nuclear distribution gene E homolog 1 (A. nidulans) (NDE1)

KIAA1627 protein (KIAA1627)

4-aminobutyrate aminotransferase (ABAT)

chromosome 8 open reading frame 59 (C8orf59)

superoxide dismutase 2, mitochondrial (SOD2)

PREDICTED: Homo sapiens similar to CG14853-PB (LOC285141)

pentatricopeptide repeat domain 2 (PTCD2)

PTPRF interacting protein, binding protein 2 (liprin beta 2) (PPFIBP2)

cyclic AMP phosphoprotein, 19 kD (ARPP-19)

TANK-binding kinase 1 (TBK1)

dystrophin (muscular dystrophy, Duchenne and Becker types) (DMD), transcript variant Dp71b

3 beta-hydroxysteroid dehydrogenase type 7

pallidin homolog (mouse) (PLDN)

gap junction protein, beta 3, 31kDa (GJB3), transcript variant 1

KIAA1715 (KIAA1715)

HIV-1 Rev binding protein (HRB)

Spermatogenesis-associated protein 4

thiopurine S-methyltransferase (TPMT)

chromosome 6 open reading frame 113 (C6orf113)

BTB (POZ) domain containing 12 (BTBD12)

synovial sarcoma, X breakpoint 3 (SSX3), transcript variant 1

Endoplasmic reticulum resident protein ERp27

Pyruvate kinase isozymes R/L

synaptojanin 2 (SYNJ2)

WD repeat domain 61 (WDR61)

breast carcinoma amplified sequence 3 (BCAS3), transcript variant 2

fibroblast growth factor 12 (FGF12)

glutamic-pyruvate transaminase (alanine aminotransferase) (GPT)

nuclear factor I/C (CCAAT-binding transcription factor) (NFIC)

LSM14A, SCD6 homolog A (S. cerevisiae) (LSM14A)

mediator complex subunit 30 (MED30)

Zinc finger protein ZXDC

UNC-112 related protein 2 (URP2)

LIM domain-binding protein 1

ankyrin repeat and KH domain containing 1 (ANKHD1), transcript variant 3

Interferon-inducible double stranded RNA-dependent protein kinase activator A

transcription factor 19 (SC1) (TCF19)

chromosome 20 open reading frame 132 (C20orf132)

v-rel reticuloendotheliosis viral oncogene homolog A, nuclear factor of kappa light polypeptide gene enhancer in B-cells 3, p65 (avian) (RELA)

Lipoma HMGIC fusion partner

mediator of RNA polymerase II transcription, subunit 8 homolog (S. cerevisiae) (MED8), transcript variant 4

chromosome 2 open reading frame 51 (C2orf51)

Uncharacterized protein C19orf57

zinc finger E-box binding homeobox 2 (ZEB2)

FK506 binding protein 3, 25kDa (FKBP3)

ELL associated factor 1 (EAF1)

LIM and cysteine-rich domains 1 (LMCD1)

runt-related transcription factor 1; translocated to, 1 (cyclin D-related) (RUNX1T1), transcript variant 1

endoglin (Osler-Rendu-Weber syndrome 1) (ENG)

fibronectin 1 (FN1)

egl nine homolog 2 (C. elegans) (EGLN2), transcript variant 1

aminoadipate aminotransferase (AADAT)

UCHL5 interacting protein (UCHL5IP)

Vesicle-trafficking protein SEC22b

Synphilin-1

secretory carrier membrane protein 3 (SCAMP3), transcript variant 2

fibroblast growth factor 12 (FGF12), transcript variant 2

Coiled-coil domain-containing protein 15

chromobox homolog 5 (HP1 alpha homolog, Drosophila) (CBX5)

ADP-ribosylation factor-like protein 6-interacting protein 4

26S protease regulatory subunit 4

hypothetical protein MGC26641 (MGC26641)

mitogen-activated protein kinase organizer 1 (MORG1), transcript variant 2

zinc finger and SCAN domain containing 18 (ZSCAN18)

melanoma antigen family B, 1 (MAGEB1), transcript variant 1

Complement factor D

coiled-coil domain containing 131 (CCDC131)

thioredoxin-like 4B (TXNL4B)

zinc finger protein-like 1 (ZFPL1)

DEAD (Asp-Glu-Ala-Asp) box polypeptide 42 (DDX42)

Histone deacetylase 7

B-cell CLL/lymphoma 7B (BCL7B)

protein tyrosine phosphatase, non-receptor type 2 (PTPN2), transcript variant 3

Aprataxin and PNK-like factor

chromodomain helicase DNA binding protein 2 (CHD2)

RAN binding protein 5 (RANBP5)

intestinal cell (MAK-like) kinase (ICK), transcript variant 1

MAP/microtubule affinity-regulating kinase 2 (MARK2), transcript variant 3

heat shock protein 90kDa beta (Grp94), member 1 (HSP90B1)

chromatin modifying protein 6 (CHMP6)

collagen, type XXIII, alpha 1 (COL23A1)

RING finger protein 219

platelet-activating factor acetylhydrolase, isoform Ib, gamma subunit 29kDa (PAFAH1B3)

Williams-Beuren syndrome chromosomal region 28 protein

lectin, galactoside-binding, soluble, 8 (galectin 8) (LGALS8)

calpain 3, (p94) (CAPN3), transcript variant 4

chromosome 12 open reading frame 39 (C12orf39)

Coiled-coil domain-containing protein 6

microsomal triglyceride transfer protein (MTTP)

homer homolog 3 (Drosophila) (HOMER3)

translin-associated factor X interacting protein 1 (TSNAXIP1)

mitochondrial ribosomal protein L16 (MRPL16), nuclear gene encoding mitochondrial protein

FLJ43980 protein (FLJ43980)

TRAF2 and NCK interacting kinase (TNIK)

chromosome 9 open reading frame 78 (C9orf78)

myocyte enhancer factor 2A (MEF2A)

feline sarcoma oncogene (FES)

proteasome (prosome, macropain) activator subunit 1 (PA28 alpha) (PSME1), transcript variant 1

mitogen-activated protein kinase 10 (MAPK10), transcript variant 1

disabled homolog 1 (Drosophila) (DAB1)

Parkinson disease 7 domain containing 1 (PDDC1)

Protein phosphatase Slingshot homolog 3

basic helix-loop-helix domain containing, class B, 9 (BHLHB9)

Z-DNA binding protein 1 (ZBP1)

zinc finger protein 333 (ZNF333)

ubiquitin protein ligase E3A (human papilloma virus E6-associated protein, Angelman syndrome) (UBE3A)

transducin (beta)-like 3 (TBL3)

tubulin folding cofactor B (TBCB)

zinc finger, MYM-type 3 (ZMYM3)

chromosome 19 open reading frame 40 (C19orf40)

Amyotrophic lateral sclerosis 2 chromosomal region candidate gene 4 protein

ankyrin repeat and zinc finger domain containing 1 (ANKZF1)

Rap guanine nucleotide exchange factor (GEF) 4 (RAPGEF4)

chromosome 8 open reading frame 22 (C8orf22)

poliovirus receptor-related 3 (PVRL3)

complexin 1 (CPLX1)

Protein arginine N-methyltransferase 6

programmed cell death 4 (neoplastic transformation inhibitor) (PDCD4)

myeloid/lymphoid or mixed-lineage leukemia (trithorax homolog, Drosophila); translocated to, 3 (MLLT3)

Protein DBF4 homolog B

protein phosphatase 1, regulatory (inhibitor) subunit 10 (PPP1R10)

Uncharacterized protein C19orf44

PHD finger protein 11 (PHF11)

v-ets erythroblastosis virus E26 oncogene homolog 1 (avian) (ETS1)

sterile alpha motif domain containing 4A (SAMD4A)

vacuolar protein sorting 35 homolog (S. cerevisiae) (VPS35)

leucine rich repeat containing 48 (LRRC48)

Intraflagellar transport protein 81 homolog

erythrocyte membrane protein band 4.9 (dematin) (EPB49)

surfeit 5 (SURF5), transcript variant a

tRNA methyltransferase 12 homolog (S. cerevisiae) (TRMT12)

zinc finger, FYVE domain containing 28 (ZFYVE28)

lectin, galactoside-binding, soluble, 3 (LGALS3)

bromodomain containing 3 (BRD3)

TBC1 domain family member 9B

chromosome 14 open reading frame 79 (C14orf79)

BCL2-associated athanogene 2 (BAG2)

family with sequence similarity 78, member A (FAM78A)

Metastasis-associated protein MTA1

serine/threonine kinase 31 (STK31)

kinesin family member C1 (KIFC1)

myocyte enhancer factor 2D (MEF2D)

zinc finger, ZZ-type containing 3 (ZZZ3)

family with sequence similarity 32, member A (FAM32A)

serine-arginine repressor protein (35 kDa) (SRrp35)

PCTAIRE protein kinase 3 (PCTK3)

RUN domain containing 3A (RUNDC3A)

epsin 1 (EPN1)

chromatin assembly factor 1, subunit B (p60) (CHAF1B)

signal transducer and activator of transcription 6, interleukin-4 induced (STAT6)

transcription factor CP2-like 1 (TFCP2L1)

holocarboxylase synthetase (biotin-(proprionyl-Coenzyme A-carboxylase (ATP-hydrolysing)) ligase) (HLCS)

Wolf-Hirschhorn syndrome candidate 2 (WHSC2)

tigger transposable element derived 1 (TIGD1)

Fas (TNFRSF6) binding factor 1 (FBF1)

Centromere protein T

XRCC6 binding protein 1 (XRCC6BP1)

sirtuin (silent mating type information regulation 2 homolog) 6 (S. cerevisiae) (SIRT6)

pim-1 oncogene (PIM1)

kinesin family member 4A (KIF4A)

DnaJ (Hsp40) homolog, subfamily C, member 8 (DNAJC8)

DTW domain containing 1 (DTWD1)

protein kinase C, alpha (PRKCA); see catalog number for detailed information on wild-type or point mutant status

chromosome 12 open reading frame 45 (C12orf45)

Disks large-associated protein 5

Serine/threonine-protein phosphatase 4 regulatory subunit 3A

host cell factor C1 regulator 1 (XPO1 dependent) (HCFC1R1), transcript variant 3

HD domain containing 3 (HDDC3)

E3 ubiquitin-protein ligase LRSAM1

synaptophysin-like 1 (SYPL1), transcript variant 2

NADH dehydrogenase (ubiquinone) 1 alpha subcomplex, 11, 14.7kDa (NDUFA11)

hepcidin antimicrobial peptide (HAMP)

RING1 and YY1 binding protein (RYBP)

casein kinase 1, gamma 1 (CSNK1G1)

Regulator of G-protein signaling 3

CSAG family, member 3A (CSAG3A)

kinesin heavy chain member 2A (KIF2A)

Butyrophilin subfamily 1 member A1

ATG4 autophagy related 4 homolog A (S. cerevisiae) (ATG4A), transcript variant 1

peroxisome proliferative activated receptor, delta (PPARD), transcript variant 2

Splicing factor 45

Regulator of G-protein signaling 7

239 antibodies for which the OD ratio of acute FT1D to sub-acute was >1.4 in Case 3

CMRF35-like molecule 2 (CD300e)

nudE nuclear distribution gene E homolog 1 (A. nidulans) (NDE1)

tumor necrosis factor (ligand) superfamily, member 13b (TNFSF13B)

kinesin family member 26A (KIF26A)

alcohol dehydrogenase 5 (class III), chi polypeptide (ADH5)

phosphoglycerate mutase 1 (brain) (PGAM1)

peptidylprolyl isomerase E (cyclophilin E) (PPIE), transcript variant 1

centaurin, alpha 2 (CENTA2)

tensin 1 (TNS1)

paroxysmal nonkinesigenic dyskinesia (PNKD), transcript variant 2

Tryptophanyl-tRNA synthetase, cytoplasmic

chromosome 9 open reading frame 78 (C9orf78)

pyruvate dehydrogenase kinase, isozyme 4 (PDK4)

ribosomal protein L26 (RPL26)

LIM domain and actin-binding protein 1

ribosomal protein S6 kinase, 70kDa, polypeptide 1 (RPS6KB1)

Disks large homolog 3

Nicotinamide mononucleotide adenylyltransferase 3

Dixin

eukaryotic translation initiation factor 2B, subunit 2 beta, 39kDa (EIF2B2)

amphiphysin (AMPH)

actinin, alpha 2 (ACTN2)

Phosphofurin acidic cluster sorting protein 2

Meis homeobox 2 (MEIS2), transcript variant f

bromodomain containing 3 (BRD3)

chromosome 11 open reading frame 51 (C11orf51)

Guanine nucleotide-binding protein subunit alpha-14

Beta-sarcoglycan

RAS guanyl releasing protein 3 (calcium and DAG-regulated) (RASGRP3)

ATP-binding cassette sub-family D member 4

proteasome (prosome, macropain) 26S subunit, non-ATPase, 9 (PSMD9)

phosphoribosyl pyrophosphate synthetase 1 (PRPS1)

HIRA interacting protein 3 (HIRIP3)

eukaryotic translation initiation factor 3, subunit G (EIF3S4)

maelstrom homolog (Drosophila) (MAEL)

Tryptophanyl-tRNA synthetase, cytoplasmic

dystrophin (muscular dystrophy, Duchenne and Becker types) (DMD), transcript variant Dp140

Hematopoietic lineage cell-specific protein

cardiotrophin-like cytokine factor 1 (CLCF1)

janus kinase and microtubule interacting protein 2 (JAKMIP2)

germinal center expressed transcript 2 (GCET2), transcript variant 2

MOB1, Mps One Binder kinase activator-like 2A (yeast) (MOBKL2A)

Putative E3 ubiquitin-protein ligase SH3RF2

family with sequence similarity 133, member B (FAM133B), transcript variant 1

uroporphyrinogen decarboxylase (UROD)

alanine-glyoxylate aminotransferase 2-like 2 (AGXT2L2)

THAP domain containing, apoptosis associated protein 1 (THAP1), transcript variant 1

elaC homolog 1 (E. coli) (ELAC1)

nuclear distribution gene C homolog (A. nidulans) (NUDC)

nucleoporin 50kDa (NUP50), transcript variant 3

CHK1 checkpoint homolog (S. pombe) (CHEK1)

RING finger and CHY zinc finger domain-containing protein 1

Hypermethylated in cancer 2 protein

Probable ATP-dependent RNA helicase DDX58

potassium voltage-gated channel, shaker-related subfamily, beta member 1 (KCNAB1), transcript variant 3

serpin peptidase inhibitor, clade B (ovalbumin), member 4 (SERPINB4)

chromosome X open reading frame 56 (CXorf56)

LIM and cysteine-rich domains 1 (LMCD1)

zinc finger protein 24 (ZNF24)

disabled homolog 1 (Drosophila) (DAB1)

general transcription factor IIB (GTF2B)

tryptophanyl-tRNA synthetase (WARS), transcript variant 3

amyloid beta (A4) precursor protein-binding, family A, member 2 binding protein (APBA2BP)

TAF6 RNA polymerase II, TATA box binding protein (TBP)-associated factor, 80kDa (TAF6), transcript variant 1

serine hydroxymethyltransferase 1 (soluble) (SHMT1), transcript variant 1

four and a half LIM domains 1 (FHL1)

KIAA1576 protein (KIAA1576)

Usher syndrome 1C (autosomal recessive, severe) (USH1C)

family with sequence similarity 80, member A (FAM80A)

interferon regulatory factor 3 (IRF3)

chromosome 9 open reading frame 9 (C9orf9)

Polycomb protein EZH2

Tetratricopeptide repeat protein 15

calcitonin gene-related peptide-receptor component protein (RCP9)

FYVE, RhoGEF and PH domain-containing protein 2

proline synthetase co-transcribed homolog (bacterial) (PROSC)

Cytosolic non-specific dipeptidase

ADP-ribosylation factor-like 8B (ARL8B)

Alcohol dehydrogenase class 4 mu/sigma chain

cold shock domain containing C2, RNA binding (CSDC2)

cysteine and glycine-rich protein 1 (CSRP1)

septin 9 (SEPT9)

amyotrophic lateral sclerosis 2 (juvenile) chromosome region, candidate 8 (ALS2CR8)

Transducin-like enhancer protein 4

CDC42 small effector 1 (CDC42SE1), transcript variant 2

disabled homolog 1 (Drosophila) (DAB1)

protein phosphatase 2, regulatory subunit B', gamma isoform (PPP2R5C), transcript variant 4

docking protein 1, 62kDa (downstream of tyrosine kinase 1) (DOK1)

nei like 2 (E. coli) (NEIL2)

chromosome 20 open reading frame 198 (C20orf198)

Small proline-rich protein 3

RNA binding motif protein 9 (RBM9)

PREDICTED: Homo sapiens hypothetical gene supported by AK075484; BC014578 (LOC339804)

General transcription factor II-I

TIMP metallopeptidase inhibitor 4 (TIMP4)

Amyotrophic lateral sclerosis 2 chromosomal region candidate gene 4 protein

butyrobetaine (gamma), 2-oxoglutarate dioxygenase (gamma-butyrobetaine hydroxylase) 1 (BBOX1)

YTH domain family, member 2 (YTHDF2)

enhancer of rudimentary homolog (Drosophila) (ERH)

KIAA0515 (KIAA0515)

amelogenin (amelogenesis imperfecta 1, X-linked) (AMELX), transcript variant 1

NDRG family member 4 (NDRG4)

family with sequence similarity 112, member A (FAM112A), transcript variant 1

guanosine monophosphate reductase (GMPR)

aminoadipate aminotransferase (AADAT)

integrin-linked kinase-associated serine/threonine phosphatase 2C (ILKAP)

mitogen-activated protein kinase kinase kinase 11 (MAP3K11)

cDNA clone IMAGE:3351130, complete cds

A.T hook DNA-binding motif-containing protein 1

chromosome 11 open reading frame 30 (C11orf30)

N-acetyltransferase 6 (NAT6)

phosphodiesterase 4D interacting protein (myomegalin) (PDE4DIP)

ADP-ribosylation factor-like 9 (ARL9)

Coiled-coil domain-containing protein 106

Bruton agammaglobulinemia tyrosine kinase (BTK)

nudix (nucleoside diphosphate linked moiety X)-type motif 21 (NUDT21)

immunoglobulin (CD79A) binding protein 1 (IGBP1)

PHD finger protein 20-like 1 (PHF20L1), transcript variant 3

FERM domain containing 8 (FRMD8)

interleukin 18 binding protein (IL18BP), transcript variant A

chromosome 9 open reading frame 37 (C9orf37)

Four and a half LIM domains protein 3

Meis homeobox 2 (MEIS2), transcript variant d

BAI1-associated protein 2 (BAIAP2)

paroxysmal nonkinesigenic dyskinesia (PNKD), transcript variant 1

tumor protein D52-like 1 (TPD52L1), transcript variant 3

aprataxin (APTX), transcript variant 4

MAM domain containing 2 (MAMDC2)

chromosome 4 open reading frame 19 (C4orf19)

splicing factor, arginine/serine-rich 12 (SFRS12), transcript variant 2

CaM kinase-like vesicle-associated (CAMKV)

TFS2-M domain-containing protein 1 (MGC17403)

matrix metallopeptidase 8 (neutrophil collagenase) (MMP8)

XIAP associated factor-1 (XAF1), transcript variant 1

WD repeat-containing protein 33

calcium/calmodulin-dependent protein kinase (CaM kinase) II alpha (CAMK2A), transcript variant 1

general transcription factor IIF, polypeptide 1, 74kDa (GTF2F1)

sorting nexin 7 (SNX7)

NADH dehydrogenase (ubiquinone) 1 beta subcomplex, 1, 7kDa (NDUFB1)

chromosome 12 open reading frame 41 (C12orf41)

upstream transcription factor 1 (USF1), transcript variant 2

WAS/WASL-interacting protein family member 1

betaine-homocysteine methyltransferase 2 (BHMT2)

lectin, galactoside-binding, soluble, 3 (LGALS3)

calcium/calmodulin-dependent protein kinase ID (CAMK1D), transcript variant 1

Twinfilin-1

Rtf1, Paf1/RNA polymerase II complex component, homolog (S. cerevisiae) (RTF1)

chromosome 1 open reading frame 165 (C1orf165)

polymerase (RNA) II (DNA directed) polypeptide F (POLR2F)

Neuronatin

UBX domain-containing protein 7

ribosomal protein L41 (RPL41), transcript variant 1

endoplasmic reticulum protein 29 (ERP29), transcript variant 1

PHD finger protein 19

Cbp/p300-interacting transactivator 1

Tigger transposable element-derived protein 1

mitogen-activated protein kinase 14 (MAPK14), transcript variant 2

Uncharacterized protein KIAA1310

zinc finger, RAN-binding domain containing 2 (ZRANB2), transcript variant 1

Galectin-3

ankyrin repeat and SOCS box-containing 9 (ASB9)

AP2 associated kinase 1 (AAK1)

Leiomodin-1

Zinc finger matrin-type protein 4

nucleolar and coiled-body phosphoprotein 1 (NOLC1)

chromosome 1 open reading frame 63 (C1orf63)

Thioredoxin reductase 1, cytoplasmic

ketohexokinase (fructokinase) (KHK)

COMM domain containing 10 (COMMD10)

inositol(myo)-1(or 4)-monophosphatase 2 (IMPA2)

zinc finger protein 333 (ZNF333)

TIP41, TOR signaling pathway regulator-like (S. cerevisiae) (TIPRL)

ferritin, light polypeptide (FTL)

GA binding protein transcription factor, beta subunit 2 (GABPB2), transcript variant gamma-2

polymerase (DNA-directed), delta 3, accessory subunit (POLD3)

Tubulin-specific chaperone cofactor E-like protein

Endophilin-B2

cDNA clone MGC:40426 IMAGE:5178085, complete cds

coiled-coil domain containing 99 (CCDC99)

DEAD (Asp-Glu-Ala-Asp) box polypeptide 43 (DDX43)

Pentatricopeptide repeat-containing protein 3, mitochondrial

sorcin (SRI), transcript variant 1

RD RNA binding protein (RDBP)

activating transcription factor 2 (ATF2)

SLAIN motif family, member 2 (SLAIN2)

StAR-related lipid transfer (START) domain containing 10 (STARD10)

Serine/threonine-protein kinase 6

microsomal triglyceride transfer protein (MTTP)

Homeobox protein TGIF2LX

family with sequence similarity 126, member B (FAM126B)

spermidine/spermine N1-acetyltransferase 2 (SAT2)

Small proline-rich protein 2G

proline-serine-threonine phosphatase interacting protein 1 (PSTPIP1)

fumarylacetoacetate hydrolase (fumarylacetoacetase) (FAH)

signal recognition particle 19kDa (SRP19)

mediator complex subunit 19 (MED19)

prolyl endopeptidase (PREP)

fibronectin type III and SPRY domain containing 1-like (FSD1L), transcript variant 1

chromosome 6 open reading frame 113 (C6orf113)

peptidylprolyl isomerase A (cyclophilin A) (PPIA)

Diphosphoinositol polyphosphate phosphohydrolase 3-alpha

hematopoietic SH2 domain containing (HSH2D)

chromodomain helicase DNA binding protein 2 (CHD2)

TCR gamma alternate reading frame protein (TARP), nuclear gene encoding mitochondrial protein, transcript variant 1

ninjurin 1 (NINJ1)

soc-2 suppressor of clear homolog (C. elegans) (SHOC2)

zinc finger, MYND-type containing 19 (ZMYND19)

zinc finger protein 650 (ZNF650)

zinc finger, CCHC domain containing 11 (ZCCHC11)

arginase, liver (ARG1)

zinc finger protein 398 (ZNF398), transcript variant 2

Adipophilin

FtsJ homolog 1 (E. coli) (FTSJ1), transcript variant 1

RAN binding protein 3 (RANBP3)

6-phosphofructo-2-kinase/fructose-2,6-biphosphatase 4 (PFKFB4)

sialic acid binding Ig-like lectin 6 (SIGLEC6)

LIM domain-binding protein 1

CDC-like kinase 4 (CLK4)

hypothetical protein FLJ23356 (FLJ23356)

Aldehyde dehydrogenase family 8 member A1

intercellular adhesion molecule 4 (Landsteiner-Wiener blood group) (ICAM4), transcript variant 1

yippee-like 1 (Drosophila) (YPEL1)

N-acetylneuraminic acid synthase (sialic acid synthase) (NANS)

Leukotriene C4 synthase

Cell growth regulator with RING finger domain protein 1

PREDICTED: Homo sapiens hypothetical LOC389081 (LOC389081)

RING finger protein 31

zinc finger, MYM-type 3 (ZMYM3)

creatine kinase, muscle (CKM)

grancalcin, EF-hand calcium binding protein (GCA)

POM121 and ZP3 fusion protein

SET and MYND domain containing 3 (SMYD3)

proteasome (prosome, macropain) subunit, alpha type, 7 (PSMA7), transcript variant 2

tripartite motif-containing 44 (TRIM44)

Upstream stimulatory factor 2

v-crk sarcoma virus CT10 oncogene homolog (avian) (CRK), transcript variant I

chromosome 9 open reading frame 150 (C9orf150)

v-crk sarcoma virus CT10 oncogene homolog (avian) (CRK), transcript variant II

phenylalanyl-tRNA synthetase, alpha subunit (FARSA)

oxysterol binding protein-like 3 (OSBPL3), transcript variant 4, mRNA.

Tripartite motif-containing protein 15

angiopoietin-like 7 (ANGPTL7)

Thioredoxin reductase 1, cytoplasmic

C-X-C motif chemokine 11

Glutaminyl-tRNA synthetase

ATP-binding cassette, sub-family F (GCN20), member 3 (ABCF3)

mitochondrial ribosomal protein L1 (MRPL1), nuclear gene encoding mitochondrial protein

hypothetical protein LOC652968 (LOC652968)

DNA-directed RNA polymerase III subunit RPC9

Glutamate decarboxylase 2

31 antibodies for which the OD ratio of acute FT1D to sub-acute was >1.4 both in Cases 1 and 3

RAN binding protein 3 (RANBP3)

chromosome 11 open reading frame 30 (C11orf30)

WAS/WASL-interacting protein family member 1

Probable ATP-dependent RNA helicase DDX58

guanosine monophosphate reductase (GMPR)

ribosomal protein S6 kinase, 70kDa, polypeptide 1 (RPS6KB1)

Adipophilin

uroporphyrinogen decarboxylase (UROD)

matrix metallopeptidase 8 (neutrophil collagenase) (MMP8)

endoplasmic reticulum protein 29 (ERP29), transcript variant 1

chromosome 1 open reading frame 63 (C1orf63)

HIRA interacting protein 3 (HIRIP3)

Serine/threonine-protein kinase 6

FERM domain containing 8 (FRMD8)

Beta-sarcoglycan

chromosome X open reading frame 56 (CXorf56)

phosphoglycerate mutase 1 (brain) (PGAM1)

nucleoporin 50kDa (NUP50), transcript variant 3

alcohol dehydrogenase 5 (class III), chi polypeptide (ADH5)

TCR gamma alternate reading frame protein (TARP), nuclear gene encoding mitochondrial protein, transcript variant 1

fibronectin type III and SPRY domain containing 1-like (FSD1L), transcript variant 1

bromodomain containing 3 (BRD3)

sorting nexin 7 (SNX7)

disabled homolog 1 (Drosophila) (DAB1)

Tetratricopeptide repeat protein 15

CMRF35-like molecule 2

centaurin, alpha 2 (CENTA2)

Galectin-3

sialic acid binding Ig-like lectin 6 (SIGLEC6)

MOB1, Mps One Binder kinase activator-like 2A (yeast) (MOBKL2A)

SLAIN motif family, member 2 (SLAIN2)

44 antibodies for which the OD ratio of acute FT1D to sub-acute was >1.4 both in Cases 1 and 2

lectin, galactoside-binding, soluble, 7 (galectin 7) (LGALS7)

EPM2A (laforin) interacting protein 1 (EPM2AIP1)

p21(CDKN1A)-activated kinase 6 (PAK6)

NECAP endocytosis associated 1 (NECAP1)

chromosome 11 open reading frame 30 (C11orf30)

ankyrin repeat and MYND domain containing 1 (ANKMY1)

WAS/WASL-interacting protein family member 1

pentatricopeptide repeat domain 2 (PTCD2)

lectin, galactoside-binding, soluble, 3 (LGALS3)

ribosomal protein S6 kinase, 70kDa, polypeptide 1 (RPS6KB1)

WD repeat domain 61 (WDR61)

lectin, galactoside-binding, soluble, 1 (galectin 1) (LGALS1)

RAN binding protein 5 (RANBP5)

fibronectin 1 (FN1)

PREDICTED: Homo sapiens hypothetical protein LOC284276 (LOC284276)

protein kinase C and casein kinase substrate in neurons 2 (PACSIN2)

Mediator of RNA polymerase II transcription subunit 29

phosphodiesterase 4D, cAMP-specific (phosphodiesterase E3 dunce homolog, Drosophila) (PDE4D)

mediator of RNA polymerase II transcription, subunit 8 homolog (S. cerevisiae) (MED8), transcript variant 4

leukocyte receptor cluster (LRC) member 1 (LENG1)

FERM domain containing 8 (FRMD8)

Beta-sarcoglycan

MAP/microtubule affinity-regulating kinase 2 (MARK2), transcript variant 3

dynein, light chain, roadblock-type 2 (DYNLRB2)

zinc finger, ZZ-type containing 3 (ZZZ3)

TBC1 domain family, member 10C (TBC1D10C)

ribosomal protein S6 kinase, 70kDa, polypeptide 1 (RPS6KB1)

bromodomain containing 3 (BRD3)

translin-associated factor X interacting protein 1 (TSNAXIP1)

RNA binding motif protein 41 (RBM41)

PREDICTED: Homo sapiens similar to CG14853-PB (LOC285141)

disabled homolog 1 (Drosophila) (DAB1)

ubiquitin protein ligase E3A (human papilloma virus E6-associated protein, Angelman syndrome) (UBE3A)

Interferon-inducible double stranded RNA-dependent protein kinase activator A

CMRF35-like molecule 2 (CD300e)

ADP-ribosylation factor-like 4A (ARL4A), transcript variant 1

sorbitol dehydrogenase (SORD)

Galectin-3

lectin, galactoside-binding, soluble, 8 (galectin 8) (LGALS8)

melanoma antigen family B, 1 (MAGEB1), transcript variant 1

Rap guanine nucleotide exchange factor (GEF) 4 (RAPGEF4)

F-box protein 21 (FBXO21)

Metastasis-associated protein MTA1

Pyruvate kinase isozymes R/L

37 antibodies for which the OD ratio of acute FT1D to sub-acute was >1.4 both in Cases 2 and 3

CMRF35-like molecule 2

nudE nuclear distribution gene E homolog 1 (A. nidulans) (NDE1)

chromosome 9 open reading frame 78 (C9orf78)

ribosomal protein S6 kinase, 70kDa, polypeptide 1 (RPS6KB1)

Disks large homolog 3

bromodomain containing 3 (BRD3)

Beta-sarcoglycan

ATP-binding cassette sub-family D member 4

eukaryotic translation initiation factor 3, subunit G (EIF3S4)

cardiotrophin-like cytokine factor 1 (CLCF1)

Hypermethylated in cancer 2 protein

LIM and cysteine-rich domains 1 (LMCD1)

tryptophanyl-tRNA synthetase (WARS), transcript variant 3

amyloid beta (A4) precursor protein-binding, family A, member 2 binding protein (APBA2BP)

Usher syndrome 1C (autosomal recessive, severe) (USH1C)

Polycomb protein EZH2

ADP-ribosylation factor-like 8B (ARL8B)

disabled homolog 1 (Drosophila) (DAB1)

Amyotrophic lateral sclerosis 2 chromosomal region candidate gene 4 protein

amelogenin (amelogenesis imperfecta 1, X-linked) (AMELX), transcript variant 1

aminoadipate aminotransferase (AADAT)

mitogen-activated protein kinase kinase kinase 11 (MAP3K11)

chromosome 11 open reading frame 30 (C11orf30)

FERM domain containing 8 (FRMD8)

WAS/WASL-interacting protein family member 1

lectin, galactoside-binding, soluble, 3 (LGALS3)

Tigger transposable element-derived protein 1

Galectin-3

zinc finger protein 333 (ZNF333)

Endophilin-B2

Pentatricopeptide repeat-containing protein 3, mitochondrial

microsomal triglyceride transfer protein (MTTP)

Homeobox protein TGIF2LX

chromosome 6 open reading frame 113 (C6orf113)

chromodomain helicase DNA binding protein 2 (CHD2)

LIM domain-binding protein 1

zinc finger, MYM-type 3 (ZMYM3)
